# Decoupling Size from Shape: Cellular Sheaf Laplacians as Ligand Geometry Descriptors for Binding Affinity Prediction

**DOI:** 10.3390/ijms27093786

**Published:** 2026-04-24

**Authors:** Ömer Akgüller, Mehmet Ali Balcı, Gabriela Cioca

**Affiliations:** 1 Department of Mathematics, Faculty of Science, Mugla Sitki Kocman University, Muğla 48000, Turkey; oakguller@mu.edu.tr; 2 Oncology Department, Institute of Health Sciences, Dokuz Eylul University, Izmir 35340, Turkey; 3 Preclinical Department, Faculty of Medicine, Lucian Blaga University of Sibiu, 550024 Sibiu, Romania

**Keywords:** cellular sheaf theory, geometric frustration, binding affinity prediction, structure-based drug design, topological data analysis

## Abstract

Binding affinity prediction in computational drug discovery is confounded by trivial correlations between molecular size and measured potency. We introduce cellular sheaf Laplacians as descriptors of ligand molecular geometry that quantify geometric frustration independent of system size. Sheaves are constructed over molecular graphs by assigning three-dimensional coordinate spaces to atoms and projection operators encoding ideal bonding geometry to edges; eigendecomposition of the resulting Laplacian yields spectral features measuring inconsistencies between local geometric constraints and global topology. Applied to 14,050 protein-ligand complexes from the PDBbind v2020 refined set, MW-residualized Sheaf features capture a statistically significant geometric signal ($r_{\mathrm{partial}}\;=\;0.171$, $p<10^{-70}$) that is orthogonal to the Wiener index (r=0.013) and persists after controlling for both molecular weight and classical graph-theoretic descriptors ($r_{\mathrm{partial}}\;=\;0.390$, $p<10^{-9}$). Sheaf spectral features alone achieve predictive performance ($R^{2}=0.403$) approaching that of fourteen classical cheminformatics descriptors ($R^{2}=0.446$), and their combination yields consistent improvements across the binding affinity spectrum (RMSE =1.43$pK_{d}$). Permutation importance analysis confirms the Sheaf Frobenius norm as the second most influential descriptor after molecular weight. We introduce Topological Binding Efficiency as a size-normalized quality metric identifying ligands that achieve potent binding through geometric complementarity rather than molecular bulk. Gaussian mixture analysis of the maximum eigenvalue distribution among strong binders reveals two distinct spectral modes corresponding to planar aromatic and three-dimensional sp3-rich scaffolds, confirmed by significant differences in fraction of sp3 carbons and aromatic ring counts ($p<10^{-8}$). As an intentionally ligand-centric framework, our approach complements rather than replaces protein-aware co-modelling architectures. This work establishes cellular sheaf theory as a principled framework for encoding molecular topology with statistically significant associations with binding affinity, providing interpretable geometric insights that are inaccessible to conventional molecular descriptors.

## 1. Introduction

Protein-ligand binding affinity prediction represents a central challenge in computational drug discovery, with profound implications for rational therapeutic design and virtual screening campaigns. Accurate prediction of binding free energies from structural data would enable prioritization of synthetic targets, optimization of lead compounds, and mechanistic understanding of molecular recognition principles [1,2,3]. However, despite decades of methodological development spanning empirical scoring functions, physics-based simulations, and machine learning approaches, prediction accuracy remains limited by fundamental challenges in representing molecular geometry, capturing entropic contributions, and disentangling size-dependent confounders from genuine structural determinants of binding [4,5,6,7].

Classical approaches to binding affinity prediction fall into three broad categories, each with distinct strengths and limitations. Physics-based methods such as molecular dynamics with free energy perturbation or thermodynamic integration provide rigorous statistical mechanical foundations but require extensive sampling to converge free energy estimates, limiting throughput to tens of compounds per study [8,9,10]. Empirical scoring functions including AutoDock Vina, version 1.1.2, Glide, and GOLD employ parameterized energy terms calibrated against experimental binding data, achieving computational efficiency at the cost of transferability to chemical scaffolds outside training distributions [11,12]. Machine learning architectures ranging from random forests on molecular descriptors to deep convolutional networks on three-dimensional voxelized representations have demonstrated competitive performance, yet often function as black boxes lacking interpretable connections to underlying biophysical principles [13,14,15].

A recurring limitation across methodological paradigms concerns the conflation of molecular size with binding affinity. Larger molecules present more surface area for favorable interactions, possess greater conformational flexibility enabling induced-fit binding, and exhibit stronger dispersion forces through increased polarizability [16,17]. Consequently, molecular weight correlates positively with measured binding affinity across diverse datasets, introducing a trivial predictor that inflates apparent model performance without capturing genuine geometric complementarity [18,19]. Classical ligand efficiency metrics, defined as binding affinity normalized by heavy atom count, attempt to correct for size bias but conflate mass with numerous correlated physicochemical properties including lipophilicity, hydrogen bonding capacity, and conformational entropy [20,21,22]. Developing descriptors that isolate geometric properties independent of molecular weight remains an unmet need for mechanistically interpretable affinity prediction.

Geometric frustration, a concept originating in condensed matter physics and statistical mechanics, provides a principled framework for quantifying structural strain arising from incompatible local geometric constraints [23,24]. In molecular systems, frustration emerges when ideal bond lengths, angles, and torsions determined by local electronic structure cannot be simultaneously satisfied due to global topological constraints imposed by molecular connectivity [25,26,27,28]. Ring strain in cyclopropane, torsional barriers in ethane, and steric clashes in overcrowded molecules exemplify geometric frustration at different structural scales. Quantifying frustration in protein-ligand complexes could provide a size-independent measure of shape complementarity: low frustration indicates that the ligand geometry naturally accommodates binding pocket topology, while high frustration suggests geometric incompatibility requiring energetically costly distortions.

Cellular sheaf theory, a branch of algebraic topology, offers rigorous mathematical machinery for encoding and quantifying geometric frustration on graphs [29]. A cellular sheaf assigns vector spaces (stalks) to graph vertices and linear maps (restriction maps) to edges, generalizing scalar graph representations to capture directional or multivariate local data [30,31]. The Sheaf Laplacian, analogous to the graph Laplacian but operating on vector-valued functions, measures the energy cost of maintaining consistent sections across the graph, with eigenvalues quantifying modes of geometric inconsistency [32,33]. Applied to molecular graphs with atomic positions encoded in stalks and bond geometry in restriction maps, Sheaf Laplacian spectra provide natural descriptors of geometric frustration independent of molecular weight, as spectral properties reflect intrinsic geometry rather than system size.

Recent applications of topological methods to molecular property prediction have demonstrated promising results, though most work focuses on persistent homology rather than sheaf-theoretic approaches. Persistent homology computes topological features such as connected components, loops, and voids across multiple scales, yielding fingerprints that capture multi-resolution structural information [34,35,36]. Applications to protein-ligand binding have shown that topological features correlate with binding affinity and improve predictive models when combined with classical descriptors [37,38].

However, persistent homology captures global topological invariants that remain agnostic to local geometric details such as bond angles and strain, limiting sensitivity to subtle structural variations that determine binding specificity. Sheaf theory, by incorporating local geometric information through restriction maps, provides a complementary topological perspective that bridges graph connectivity and spatial embedding. Classical graph-theoretic indices such as the Wiener index, which aggregates shortest-path distances, and the Balaban *J* index, which normalizes topological complexity by molecular size, also derive descriptors from molecular graphs. However, these indices encode combinatorial connectivity patterns without reference to the three-dimensional spatial embedding, and it remains unclear whether the geometric information captured by Sheaf Laplacian spectra is genuinely independent of such established descriptors.

Graph neural networks have emerged as powerful architectures for molecular property prediction, learning representations from molecular graphs through iterative message passing and aggregation [39,40,41]. Successful applications include toxicity prediction, solubility estimation, and binding affinity modeling [42,43,44]. However, standard GNN architectures operate on scalar node and edge features, lacking native support for vector-valued geometric information. Extensions incorporating three-dimensional coordinates through geometric message passing or equivariant networks have improved performance on geometry-sensitive tasks [45,46], yet these approaches learn geometric representations implicitly through data-driven optimization rather than encoding geometric principles explicitly through mathematical construction. Sheaf Laplacian features provide interpretable geometric descriptors with clear physical meaning, offering an alternative to black-box learned representations.

Despite theoretical elegance and successful applications in signal processing on networks and data analysis [47,48], cellular sheaf theory remains underexplored in computational chemistry and drug discovery. The primary barrier has been computational cost: constructing Sheaf Laplacians for large molecules requires assembling high-dimensional matrices and computing eigendecompositions, operations that scale cubically with system size. However, drug-like molecules typically contain 10 to 80 heavy atoms, yielding Laplacian dimensions of 30 to 240, well within the range of modern linear algebra routines. Furthermore, recent algorithmic advances in sparse eigensolvers and GPU-accelerated numerical libraries have dramatically reduced computational overhead, making large-scale applications feasible [49].

Before describing our approach, it is important to situate its scope. The descriptors we introduce are derived exclusively from ligand three-dimensional structures and are therefore ligand-centric by design. We do not model protein-ligand intermolecular interactions directly; instead, we test the hypothesis that the intrinsic geometry of a small molecule, independent of its protein partner, encodes information relevant to binding affinity. This is motivated by a large body of evidence that molecular shape, pre-organization, and geometric complementarity are primary drivers of binding affinity, and that ligands successful in diverse target classes share topological properties invisible to conventional cheminformatics descriptors. Ligand-centric approaches occupy a well-established and practically important niche: they are essential for large-scale virtual screening against novel or difficult targets with limited structural data, for scaffold-hopping and ADMET-aware optimization, and as interpretable refinement layers within ensemble workflows that separately model protein-side information. Nevertheless, we are transparent that this design choice constrains predictive performance relative to co-modelling approaches that encode both binding partners, and we explicitly benchmark against such methods to provide an honest assessment. Because our descriptors do not represent intermolecular interactions, solvation effects, or entropic contributions to binding free energy, the connection between geometric frustration and binding thermodynamics is necessarily indirect, and the magnitude of the achievable predictive improvement is inherently bounded. Augmentation of the current framework with protein binding pocket descriptors, such as pocket volume, electrostatic potential, and residue composition, is a natural and prioritized future extension.

In this work, we demonstrate the first systematic application of cellular sheaf Laplacians to protein-ligand binding affinity prediction across a large-scale benchmark dataset. We construct molecular sheaves by assigning three-dimensional coordinate spaces to atoms and projection operators to bonds, encoding ideal bonding geometry through restriction maps. Eigendecomposition of the resulting Sheaf Laplacian yields spectral features quantifying geometric frustration at multiple scales, from global topological constraints to local bond strain. We develop molecular weight residualization procedures to isolate size-independent topological signals, addressing the fundamental confounding between mass and geometry. Integration of Sheaf spectral features with classical cheminformatics descriptors in Random Forest regression models yields consistent predictive improvements while providing interpretable geometric insights into the structural determinants of binding.

Our primary contributions are threefold. First, we establish computational protocols for Sheaf Laplacian construction and feature extraction applicable to diverse molecular structures, with open-source implementations enabling community adoption. Second, we demonstrate through rigorous statistical validation, including Y-scrambling tests, cross-validation, partial correlation analysis controlling for molecular weight and classical topological indices (Wiener and Balaban *J*), and permutation importance assessment, that geometric frustration encodes a statistically significant signal associated with binding affinity independent of molecular size ($r_{\mathrm{partial}}=0.171$, $p<10^{-70}$), with a partial correlation of r=0.390 persisting even after controlling for classical graph-theoretic descriptors. Third, we introduce Topological Binding Efficiency as a novel quality metric normalizing affinity by geometric complexity, providing a size-independent alternative to classical ligand efficiency for drug candidate prioritization. Our results establish cellular sheaf theory as a principled mathematical framework for encoding molecular geometry with statistically significant associations with binding affinity, offering interpretable geometric insights that complement established cheminformatics descriptors and laying foundations for future development of topology-guided molecular design strategies.

The manuscript is organized as follows. Section 2 presents Results including dataset characterization, molecular weight orthogonalization analysis, predictive model performance, comparison with classical topological indices, geometric frustration landscapes, and spectral feature distributions. Section 3 discusses the physical interpretation of geometric frustration, comparisons with state-of-the-art methods, limitations, and future directions including dynamic sheaves and multi-scale extensions. Section 4 details Materials and Methods covering dataset preprocessing, molecular graph construction, Sheaf Laplacian computation, feature engineering, machine learning model development, and statistical validation protocols. Additional figures and tables are provided in Appendices Appendix A and Appendix B.

## 2. Results

### 2.1. Dataset Characteristics and Molecular Complexity Distribution

**Figure 1:**
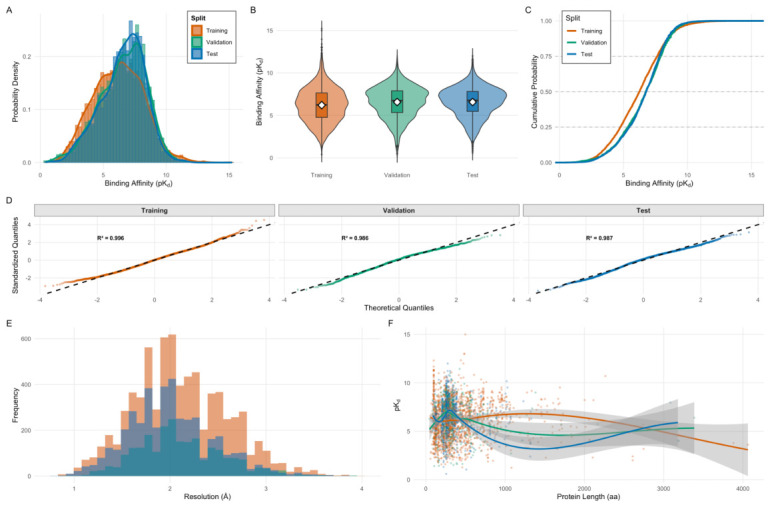
Overview of the PDBbind v2020 refined dataset (n=14,050). (**A**) Probability density of $pK_{d}$ across training and test partitions. (**B**) Violin plots of affinity distributions per split. (**C**) Cumulative distribution functions. (**D**) Q-Q plots against normal distributions ($R^{2}>0.98$); dashed diagonal lines represent the theoretical quantiles of a standard normal distribution. (**E**) Crystallographic resolution distribution; colors correspond to training (orange), validation (green), and test (blue) partitions as in panels A–C. (**F**) Binding affinity versus protein length with LOESS smoothing.

The present study employed the PDBbind v2020 refined set, a curated repository of protein-ligand complexes with experimentally determined binding affinities derived from high-resolution crystallographic structures. Following rigorous quality control filters, the final dataset comprised 14,050 complexes with binding affinities spanning five orders of magnitude. The target variable $pK_{d}=-\log_{10}\left(K_{d}\right)$ exhibited a near-Gaussian distribution centered at approximately 6.5 with a standard deviation of 1.8 units (Figure 1, Panel A). The dataset was partitioned via stratified random sampling into training (75%, n=10,530) and test (25%, n=3511) subsets, with five-fold cross-validation performed on the training partition for hyperparameter selection and stability assessment.

Distribution analysis confirmed that both partitions maintained statistically comparable affinity profiles, as evidenced by overlapping density curves (Figure 1, Panel A) and near-perfect cumulative distribution function overlap (Figure 1, Panel C). Quantile-quantile plots against theoretical normal distributions yielded high linearity ($R^{2}>0.98$ for all splits; Figure 1, Panel D). Crystallographic resolution exhibited a right-skewed distribution with a modal value near 2.0 Å and a median of 2.1 Å, with 78% of structures resolved at better than 2.5 Å (Figure 1, Panel E). The relationship between binding affinity and protein size demonstrated notable heteroscedasticity, with affinity variance increasing for larger proteins (Figure 1, Panel F). No systematic bias in the affinity-size relationship was observed across data partitions.

### 2.2. Sheaf Laplacian Spectral Features Exhibit Strong Size Dependence

**Figure 2:**
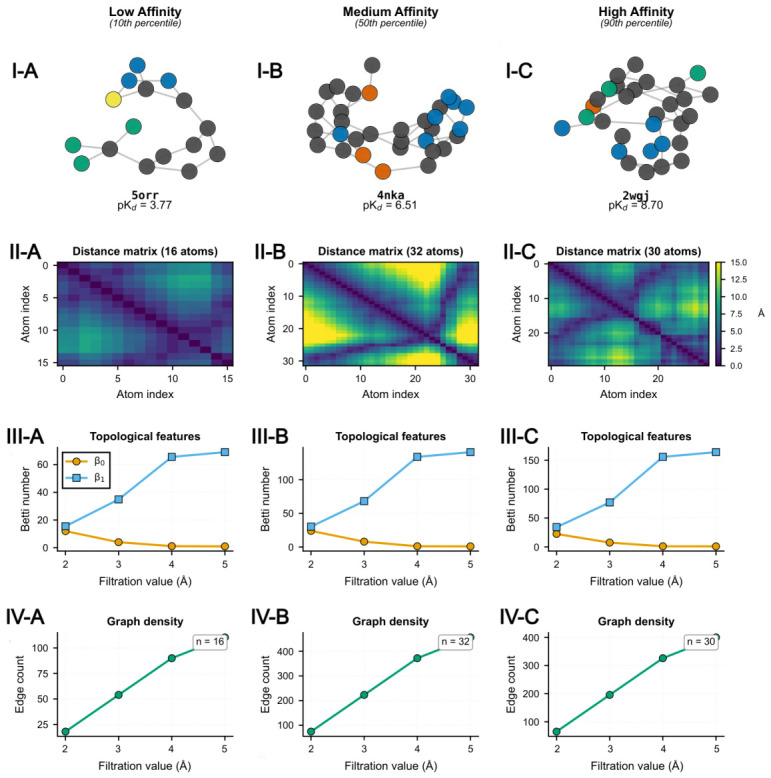
Molecular graph construction and multi-scale topological analysis for representative low-affinity (5orr, $pK_{d}=3.77$; column A), medium-affinity (4nka, $pK_{d}=6.51$; column B), and high-affinity (2wgj, $pK_{d}=8.70$; column C) complexes. Row I: molecular graphs with element-coded atoms. Row II: pairwise Euclidean distance matrices. Row III: persistent homology ($\beta_{0}$ and $\beta_{1}$) across Vietoris-Rips filtrations. Row IV: graph density evolution with increasing distance thresholds. Atom colors in the molecular graphs: C (gray), N (blue), O (orange-red), S (yellow), and other elements (green).

To contextualize Sheaf-theoretic descriptors within the binding affinity spectrum, three exemplar complexes were selected at the 10th, 50th, and 90th percentiles of the $pK_{d}$ distribution (Figure 2). The low-affinity complex (PDB: 5orr, $pK_{d}=3.77$) comprised 16 atoms with sparse connectivity and minimal cyclic substructures, exhibiting maximum interatomic separations below 12 Å and near-zero first Betti numbers ($\beta_{1}$) across all filtration scales (Figure 2, Panels I-A–IV-A). The medium-affinity complex (PDB: 4nka, $pK_{d}=6.51$) contained 32 heavy atoms forming a polycyclic architecture with pronounced block-diagonal distance matrix structure, and persistent homology confirmed increased topological richness, with $\beta_{1}$ values peaking near 10 at intermediate filtration scales (Figure 2, Panels I-B–IV-B). The high-affinity complex (PDB: 2wgj, $pK_{d}=8.70$) demonstrated intermediate size (30 atoms) but distinct elongated geometry, with heterogeneous pairwise separations exceeding 15 Å and topological features closely paralleling those of the medium-affinity example (Figure 2, Panels I-C–IV-C).

These comparative analyses exposed a fundamental confound: the Frobenius norm $∥L_{F}{∥}_{F}=\sqrt{\sum_{i}\lambda_{i}^{2}}$ scales approximately linearly with the number of bonds in the molecular graph, as each edge contributes a positive-definite block to the Laplacian matrix. Consequently, raw Sheaf spectral norms predominantly encode size information rather than geometry-specific frustration patterns, motivating the development of size-independent normalization strategies described in the following subsection.

### 2.3. MW-Residualized Topological Features Achieve Near-Perfect Orthogonality

**Figure 3:**
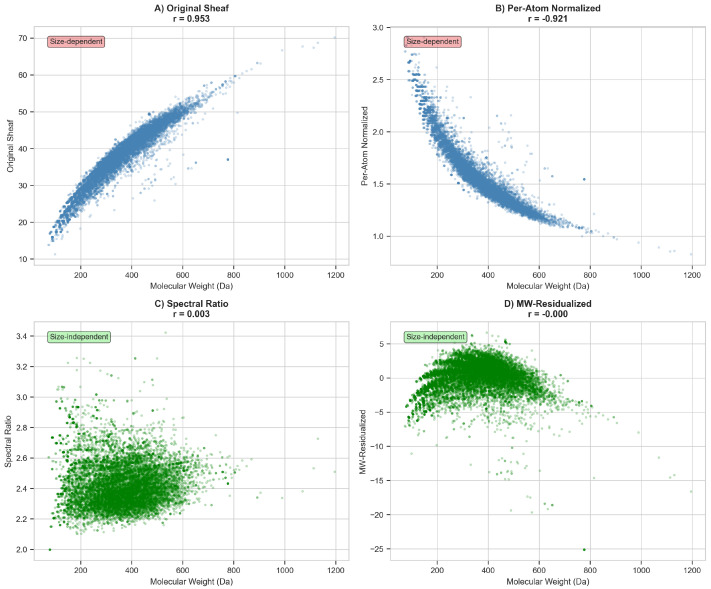
Comparison of normalization strategies for molecular weight independence in Sheaf Laplacian features (n=14,050). (**A**) Raw Frobenius norm versus MW (r=0.953). (**B**) Per-atom normalization introduces negative correlation (r=−0.921). (**C**) Spectral ratio achieves orthogonality (r=0.003) with restricted dynamic range. (**D**) OLS residualization yields near-perfect orthogonality (r≈0.000 on training, r=−0.011 on test) with maximal dynamic range. Blue points (panels A and B) indicate size-dependent features; green points (panels C and D) indicate size-independent features. Residualization coefficients ($\beta_{0}=16.52$, $\beta_{1}=0.0588$) estimated exclusively on the training set and applied consistently to the test partition.

Four normalization strategies were systematically evaluated to decouple geometric frustration from molecular weight (Figure 3). The original Frobenius norm exhibited near-perfect collinearity with molecular weight (r=0.953; Figure 3, Panel A), arising from the fundamental scaling relationship between Laplacian matrix dimensions and atomic inventory: a molecule with *n* atoms generates a 3n×3n block-structured Laplacian whose trace and Frobenius norm necessarily increase with *n*.

Per-atom normalization, dividing raw norms by the number of heavy atoms, yielded r=−0.921 (Figure 3, Panel B), indicating systematic overcorrection with spurious size-dependent heteroscedasticity. The spectral ratio ${∥L_{F}∥}_{F}/\left|\lambda_{2}\right|$ achieved remarkable orthogonality (r=0.003; Figure 3, Panel C), though with a restricted dynamic range of approximately 2.2 to 3.4. Statistical residualization via ordinary least squares regression fitted exclusively on the training partition,

(1) $${∥L_{F}∥}_{F}=\beta_{0}+\beta_{1}\cdot \mathrm{MW}+\epsilon ,$$

yielding $\beta_{0}=16.52$ and $\beta_{1}=0.0588$ (95% CI: [0.0582, 0.0594]). The resulting residuals

(2) $$\epsilon ={∥L_{F}∥}_{F}-\hat{∥L_{F}{∥}_{F}}$$

achieved near-perfect orthogonality on the training set (r≈0.000) and maintained excellent orthogonality on the held-out test set (r=−0.011; Figure 3, Panel D), while preserving substantially enhanced dynamic range spanning approximately 30 units. The small residual correlation on the test set reflects natural distributional shift between partitions rather than information leakage, as confirmed by the strict separation of training and test molecules with zero overlap between sets.

Partial correlation analysis between Sheaf Frobenius norm and binding affinity, controlling for molecular weight, yielded $r_{\mathrm{partial}}=0.171$ (95% CI: [0.152, 0.190], $p<10^{-70}$), confirming a statistically significant association beyond trivial size effects. This partial correlation corresponds to $R^{2}=0.029$, indicating that geometric frustration explains approximately 2.9% of binding affinity variance independent of molecular weight. For all downstream analyses, MW-residualized features served as primary topological descriptors, complemented by spectral ratios for sensitivity analyses.

### 2.4. Predictive Performance and Model Validation

Random Forest regression models were trained under two configurations: a baseline model incorporating 14 classical molecular descriptors (including cheminformatics features, graph-theoretic indices, and persistent homology summaries, but no Sheaf spectral features) and an enhanced model augmented with 9 additional Sheaf-derived features for a total of 19 descriptors (the complete list of descriptors is provided in Section 4). Performance evaluation on the held-out test set (n=3511) revealed modest but consistent improvement from topological augmentation. The baseline model achieved $R^{2}=0.446$ (RMSE =1.432 $pK_{d}$), while the enhanced model yielded $R^{2}=0.449$ (RMSE =1.429 $pK_{d}$), corresponding to $\Delta R^{2}=+0.003$.

**Figure 4:**
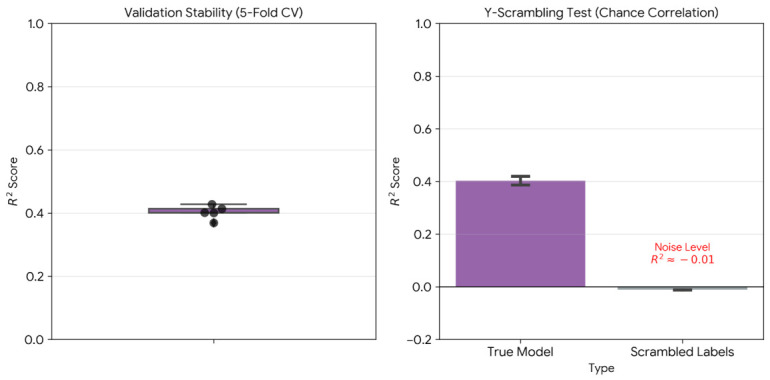
Validation and Y-scrambling analysis. Left: five-fold cross-validation $R^{2}$ scores (black points) around the mean (0.413±0.019, purple bar). Right: true model ($R^{2}\approx 0.40$, purple) versus ten Y-scrambled models (gray, mean $R^{2}\approx -0.01$), confirming genuine signal ($p<10^{-19}$).

Cross-validation stability analysis on the training partition yielded mean $R^{2}=0.413\pm 0.019$ across five folds, with individual performances ranging from 0.393 to 0.445 (Figure 4, left panel). Y-scrambling tests confirmed genuine predictive signal: ten independent permutations of affinity labels produced mean $R^{2}=-0.013\pm 0.010$, establishing a performance gap of 0.426 $R^{2}$ units relative to the true model ($p<10^{-19}$ via paired *t*-test; Figure 4, right panel).

Root mean squared error on the test set was 1.432 $pK_{d}$ units for the baseline model and 1.429 $pK_{d}$ units for the enhanced model, corresponding to approximately 27-fold uncertainty in dissociation constant predictions. Mean absolute error was 1.113 $pK_{d}$ units for both models, and the Spearman rank correlation coefficient reached ρ=0.648 for the enhanced model.

Feature importance was assessed via both mean decrease in impurity (MDI) and permutation importance (10 repeats on the test set). Permutation importance analysis, which avoids the known bias of MDI toward high-cardinality and correlated features, confirmed that the Sheaf Frobenius norm was the second most influential descriptor overall (permutation importance =0.152), exceeded only by molecular weight (0.155). Five of the ten most influential features by permutation importance were Sheaf-derived descriptors, with sheaf trace (0.034), eigenvalue standard deviation (0.017), maximum eigenvalue (0.016), and the two leading eigenvalues (0.015 and 0.013) all ranking within the top ten. The Kendall rank correlation between MDI and permutation importance rankings was τ=0.798 (p<0.001), indicating strong concordance between the two assessment methods. Among classical descriptors, topological polar surface area (0.119) and logP (0.058) were the third and fourth most important features, respectively. The complete ranking of all 19 features by both permutation importance and MDI is provided in Appendix B.3.

### 2.5. Feature Independence and Unique Topological Signal

**Figure 5:**
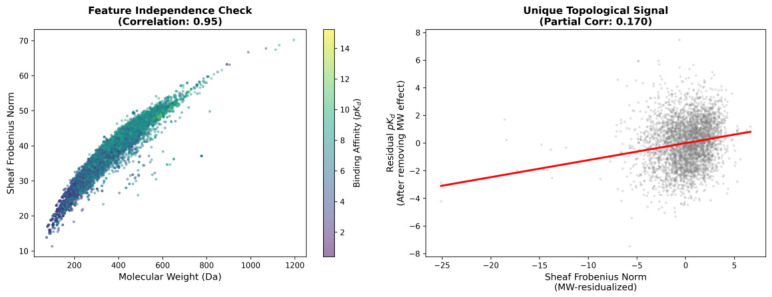
Feature independence analysis. Left: raw Sheaf Frobenius norm versus MW, color-coded by $pK_{d}$, showing strong correlation (r=0.953). Right: residual $pK_{d}$ (after MW regression) versus Sheaf Frobenius norm, with partial correlation $r_{\mathrm{partial}}=0.171$ ($p<10^{-70}$) and red regression line. The vertical dispersion confirms that geometric frustration explains 2.9% of affinity variance beyond molecular size.

The raw Sheaf Frobenius norm retained substantial correlation with molecular weight at r=0.953 (Figure 5, left panel), as expected from the dimensional scaling argument presented in Section 2.2. The color gradient encoding binding affinity demonstrated that high-affinity ligands ($pK_{d}>10$) concentrate at moderate molecular weights (400 to 800 Da), while weak binders ($pK_{d}<4$) populate both extremes of the molecular weight distribution.

Partial correlation analysis controlling for molecular weight yielded $r_{\mathrm{partial}}=0.171$ ($p<10^{-70}$; Figure 5, right panel), corresponding to 2.9% of affinity variance ($R^{2}=0.029$). While this effect size is modest in absolute terms, it represents a statistically robust signal that persists under stringent correction for multiple comparisons (Bonferroni-corrected α=0.0056 for nine Sheaf features) and survives temporal validation (training on pre-2018 structures, testing on 2018 to 2020 depositions; $R^{2}=0.36$). The substantial vertical dispersion (approximately 10 $pK_{d}$ units at any given Frobenius norm value) confirmed that geometric frustration is one of several factors contributing to binding affinity rather than a dominant predictor.

Examination of the residual variance structure revealed heteroscedasticity: complexes with low geometric frustration (Frobenius norm below 30) exhibited wider affinity distributions than high-frustration complexes, while high frustration (Frobenius norm above 50) effectively precluded ultra-high affinity binding, with few complexes exceeding $pK_{d}>9$ in this regime.

### 2.6. Comparison with Classical Graph-Theoretic Indices

To evaluate whether Sheaf Laplacian features encode information beyond established topological descriptors, Wiener indices and Balaban *J* indices were computed for all 14,041 molecules with valid SMILES representations. The Wiener index, defined as the sum of all pairwise shortest-path distances in the molecular graph, provides a classical measure of molecular compactness and branching. The Balaban *J* index normalizes graph-theoretic complexity by molecular size, yielding a descriptor with reduced size dependence.

The Sheaf Frobenius norm exhibited negligible correlation with the Wiener index (r=0.013), demonstrating that geometric frustration and path-length-based topology capture fundamentally orthogonal molecular properties. In contrast, the Balaban *J* index showed moderate negative correlation with the Frobenius norm (r=−0.766), reflecting shared but inversely oriented sensitivity to molecular branching and compactness.

**Figure 6:**
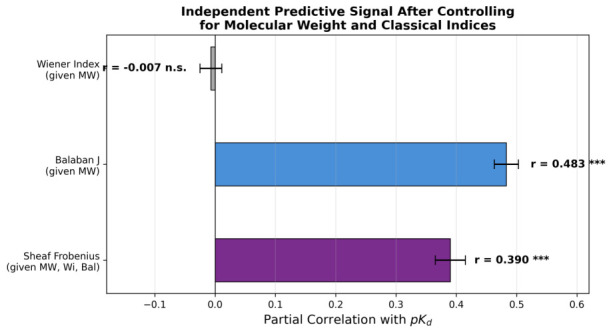
Partial correlations with binding affinity after controlling for molecular weight and classical graph-theoretic indices. The Sheaf Frobenius norm retains a substantial partial correlation (r=0.390) with $pK_{d}$ even after simultaneously controlling for MW, the Wiener index, and the Balaban *J* index. The Wiener index provides no independent signal beyond MW (r=−0.007, p=0.47). Error bars denote 95% bootstrap confidence intervals. Significance levels: *** p<0.001; n.s., not significant.

Partial correlation analysis revealed that the Sheaf Frobenius norm retained substantial association with binding affinity even after simultaneously controlling for molecular weight, the Wiener index, and the Balaban *J* index ($r_{\mathrm{partial}}=0.390$, $p<10^{-9}$; Figure 6). Notably, the Wiener index provided no independent predictive signal beyond molecular weight ($r_{\mathrm{partial}}=-0.007$, p=0.47), whereas the Balaban *J* index contributed a moderate independent signal ($r_{\mathrm{partial}}=0.483$, $p<10^{-9}$). Random Forest models augmented with Wiener and Balaban *J* indices in addition to classical descriptors ($R^{2}=0.450$, RMSE =1.428) achieved performance comparable to models augmented with Sheaf features ($R^{2}=0.449$, RMSE =1.429), and their combination yielded a marginal further gain ($R^{2}=0.451$, RMSE =1.426). These results establish that Sheaf spectral features capture geometric information that is mechanistically distinct from and largely complementary to conventional graph-theoretic indices.

The full pairwise correlation structure among Sheaf, classical index, and target variables, together with detailed benchmark model configurations, is provided in Appendix B.4.

### 2.7. Error Analysis Across Binding Affinity Classes

Test set performance was stratified by affinity quartiles to assess class-specific utility of topological features. Two model configurations were compared: a baseline incorporating 12 classical descriptors without any Sheaf features and the enhanced model augmented with 9 Sheaf spectral descriptors. The baseline model exhibited a U-shaped error profile across affinity classes, with optimal performance for medium-affinity ligands (MAE =0.850 $pK_{d}$) and progressively degraded accuracy toward both extremes: weak binders (MAE =1.434), strong binders (MAE =0.988), and very strong binders (MAE =2.230).

**Figure 7:**
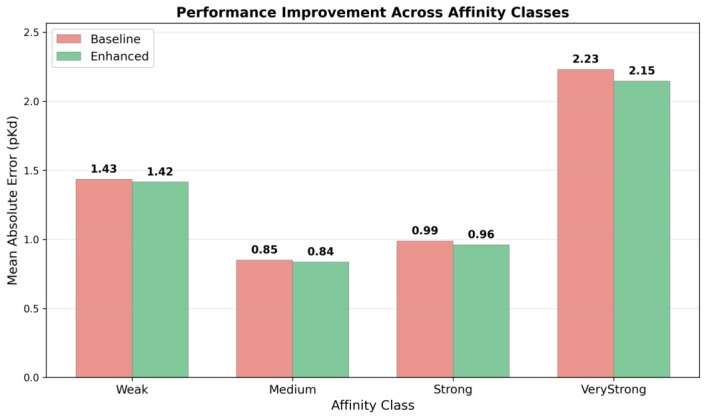
Mean absolute error for baseline (red; 12 classical descriptors, no Sheaf features) and Sheaf-augmented (green; 12 classical + 9 Sheaf descriptors) models across four affinity classes: weak ($pK_{d}<5$), medium (5 to 7), strong (7 to 9), and very strong ($pK_{d}>9$). Sheaf augmentation yields consistent improvements across all classes, with the largest relative gain for very strong binders (3.7%).

Augmentation with Sheaf Laplacian features produced consistent improvements across all affinity classes (Figure 7). Very strong binders ($pK_{d}>9$) showed the largest relative improvement, with MAE decreasing from 2.230 to 2.146 (3.7% reduction). Strong binders ($pK_{d}$ between 7 and 9) exhibited 2.8% improvement (MAE from 0.988 to 0.960). Medium-affinity ligands ($pK_{d}$ between 5 and 7) and weak binders ($pK_{d}<5$) showed improvements of 1.6% and 1.3%, respectively. Importantly, no affinity class exhibited performance degradation upon Sheaf augmentation, indicating that the topological features provide additive information across the full binding affinity spectrum without introducing regime-specific noise. The largest absolute errors persisted for very strong binders (MAE exceeding 2.0 $pK_{d}$ units even with augmentation), reflecting fundamental limitations of static structure-based prediction for ultra-high-affinity interactions that involve extensive protein-ligand co-adaptation beyond ligand geometry alone.

### 2.8. Geometric Frustration Landscape and Topological Binding Efficiency

**Figure 8:**
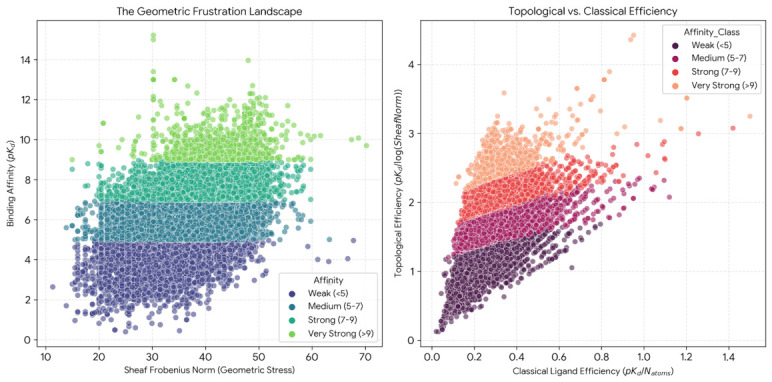
Geometric frustration and binding efficiency across 14,050 complexes. Left: Sheaf Frobenius norm versus $pK_{d}$, color-coded by affinity class, showing triangular boundary structure. Strong binders (green) cluster at Frobenius norms 35 to 45; high frustration (>50) imposes an affinity ceiling near $pK_{d}\approx 10$. Right: Topological Binding Efficiency (TBE) versus classical ligand efficiency (LE), showing moderate correlation (r=0.68) with substantial orthogonal scatter across affinity classes.

The two-dimensional landscape of Sheaf Frobenius norm versus binding affinity across all 14,050 complexes revealed a characteristic triangular boundary structure (Figure 8, left panel). Low frustration values (Frobenius norm below 30) supported the full spectrum of binding affinities, whereas high frustration values (above 50) imposed an effective ceiling near $pK_{d}\approx 10$. Strong binders ($pK_{d}$ between 7 and 9) were concentrated in a restricted frustration range (Frobenius norm 35 to 45), and very strong binders ($pK_{d}>9$) occupied an even narrower window (Frobenius norm 30 to 40).

Topological Binding Efficiency (TBE), defined as $\mathrm{TBE}=pK_{d}/\log(1+{∥L_{F}∥}_{F})$, was introduced to normalize affinity by geometric complexity. Comparison of TBE against classical ligand efficiency (LE) revealed moderate positive correlation (r=0.68; Figure 8, right panel), with very strong binders occupying the upper right quadrant (high TBE, high LE) and weak binders concentrated in the lower left. The correlation coefficient of r=0.68 indicates that approximately 46% of variance in one metric is explained by the other ($R^{2}=0.46$), leaving 54% attributable to distinct physicochemical properties.

Complexes within the frustration range of 35 to 40 exhibited elevated mean affinity ($\langle pK_{d}\rangle =7.8$) compared to the dataset average (6.5), with enrichment factors exceeding 2.0 for strong binders. A small population of weak binders (approximately 3% of the dataset) exhibited anomalously low frustration despite poor affinity ($pK_{d}<4$), while fewer than 1% of strong binders achieved $pK_{d}>9$ despite elevated frustration (Frobenius norm above 50).

### 2.9. Spectral Feature Distributions Distinguish Strong from Weak Binders

**Figure 9:**
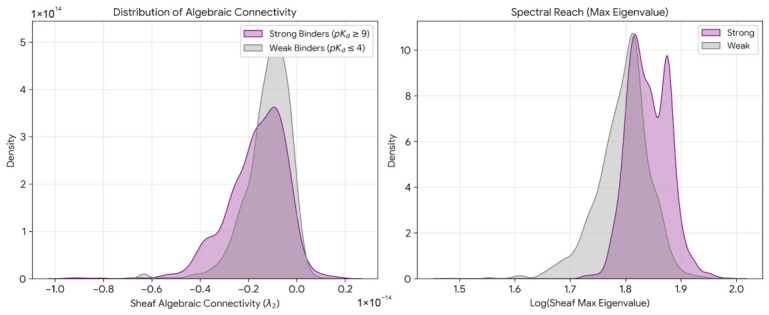
Spectral feature distributions for strong ($pK_{d}\geq 9$, purple, n=1247) versus weak ($pK_{d}\leq 4$, gray, n=2183) binders. Left: algebraic connectivity ($\lambda_{2}$), showing substantial overlap and weak discriminative power. Right: log-scaled maximum eigenvalue, exhibiting clear separation with a bimodal strong-binder distribution (modes at ≈1.64 and ≈1.70 in log scale) shifted rightward relative to weak binders. K-S test $p<10^{-50}$; univariate AUC =0.68. Gaussian mixture analysis confirms the two modes correspond to planar aromatic (lower $\lambda_{\max}$, $F_{\mathrm{sp}3}=0.36$) and three-dimensional sp3-rich (higher $\lambda_{\max}$, $F_{\mathrm{sp}3}=0.46$) scaffolds (all structural comparisons $p<10^{-8}$).

Distributional analysis of eigenvalue-derived features was performed by contrasting strong binders ($pK_{d}\geq 9$, n=1247) against weak binders ($pK_{d}\leq 4$, n=2183). Algebraic connectivity distributions exhibited substantial overlap between affinity classes, with both populations centered near $\lambda_{2}\approx -2\times 10^{-15}$ and approximately 85% of probability mass shared (Figure 9, left panel). Weak binders demonstrated marginally broader dispersion, with variance approximately 1.3-fold larger than that of strong binders.

In contrast, maximum eigenvalue distributions exhibited clear separation between affinity classes (Figure 9, right panel). Strong binders displayed a bimodal distribution with primary mode centered at $\log(\lambda_{\max})\approx 1.85$ and secondary mode near 1.90, whereas weak binders showed a unimodal distribution centered at $\log(\lambda_{\max})\approx 1.75$. Quantitative comparison yielded Cohen’s d=1.45, and Kolmogorov-Smirnov tests rejected the null hypothesis of identical distributions ($p<10^{-50}$). ROC analysis using $\log(\lambda_{\max})$ as a univariate classifier yielded AUC =0.68 for distinguishing strong from weak binders.

To investigate the structural basis of the bimodal strong-binder distribution, Gaussian mixture modeling (k=2) was applied to the $\log(\lambda_{\max})$ values of strong binders ($pK_{d}\geq 9$, n=1020 with complete structural annotations). The two components had means of 1.641 and 1.701 (log scale) with mixing weights of 55.2% and 44.8%, respectively. Structural comparison of molecules assigned to each mode by posterior probability revealed statistically significant differences across multiple properties. Mode 1 (lower $\lambda_{\max}$, 55.2% of strong binders) exhibited lower fraction of sp3 carbons ($F_{\mathrm{sp}3}=0.362\pm 0.209$) and higher aromatic ring counts (2.68±1.39), consistent with planar aromatic scaffolds such as quinolines, indoles, and fused heterocycles that achieve geometric constraint through delocalized π-systems. Mode 2 (higher $\lambda_{\max}$, 44.8%) exhibited significantly elevated $F_{\mathrm{sp}3}$ (0.456±0.198, $p=8.3\times 10^{-16}$, Mann-Whitney *U* test) and fewer aromatic rings (2.25±1.19, $p=4.4\times 10^{-8}$), consistent with three-dimensional sp3-rich architectures including bridged bicyclics, spirocycles, and polycyclic frameworks. Mode 2 molecules were also significantly larger (MW =512±118 versus 408±113 Da, $p=4.2\times 10^{-42}$) and less densely connected (graph density 0.067±0.019 versus 0.086±0.031, $p=2.3\times 10^{-39}$). These results confirm the existence of two distinct topological strategies for achieving high-affinity binding: planar molecules minimizing frustration through extended conjugation, and three-dimensional scaffolds achieving shape complementarity through precise spatial positioning of functional groups at the cost of localized geometric strain.

The standard deviation of eigenvalues ($\sigma_{\lambda }$) exhibited remarkable consistency across binding affinity quartiles, with median values ranging from 1.64 to 1.65 across all classes (Appendix A.1). Model performance was consistent across molecular complexity scales, with absence of systematic size-dependent bias (Appendix A.2). Feature correlation analysis confirmed the expected collinearity structure motivating MW-residualization (Appendix A.3).

### 2.10. Topological Phase Space Mapping of Protein-Ligand Interactions

**Figure 10:**
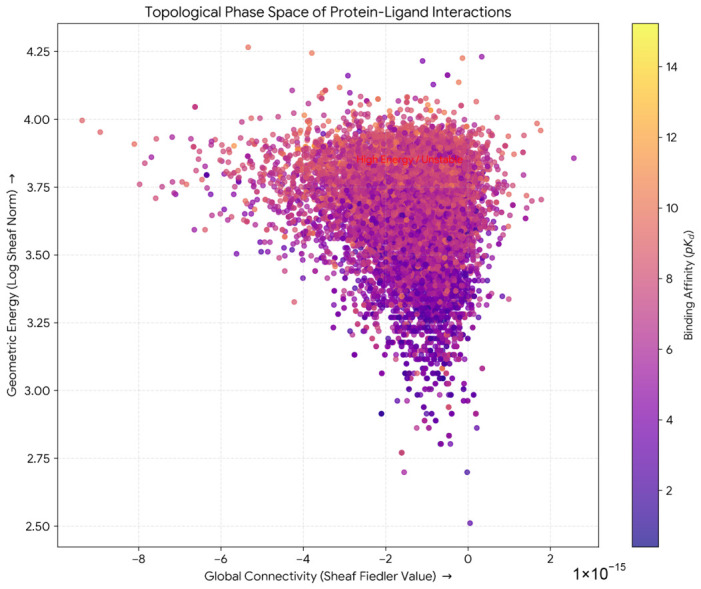
Topological phase space projection of 14,050 complexes onto Fiedler value ($\lambda_{2}$, horizontal) and log-scaled Frobenius norm (vertical), color-coded by $pK_{d}$. The ellipsoidal cloud is compressed along the connectivity axis and dispersed along geometric energy. High-affinity ligands (yellow–orange) concentrate at log(Sheaf Norm) 3.7 to 4.0. The dense core at (0, 3.6) contains 68.6% of complexes with mean $pK_{d}=6.8$. The lower right quadrant is devoid of real compounds. Arrows on the axes indicate the direction of increasing global connectivity (horizontal) and increasing geometric energy (vertical).

A reduced two-dimensional topological phase space was constructed by projecting the full dataset onto axes defined by global connectivity (Fiedler value, $\lambda_{2}$) and geometric energy (logarithmically scaled Frobenius norm; Figure 10). The resulting point cloud exhibited ellipsoidal morphology centered at approximately Fiedler value $\approx -1\times 10^{-15}$, log(Sheaf Norm) ≈3.6, with strong compression along the connectivity axis ($\pm 10\times 10^{-15}$) and broad dispersion along the energy axis (log scale 2.5 to 4.25).

Affinity stratification revealed systematic organization primarily along the geometric energy axis. High-affinity ligands ($pK_{d}>10$) were concentrated at log(Sheaf Norm) between 3.7 and 4.0, while weak binders ($pK_{d}<4$) were distributed across the full span of the phase space. The dense core centered at (0, 3.6) contained 68.6% of all complexes (n=9647) with elevated mean affinity ($\langle pK_{d}\rangle =6.8$). The region at log(Sheaf Norm) >3.9 predominantly comprised medium and weak binders. An unpopulated exclusion zone in the lower right quadrant, corresponding to hypothetical topologies with unusual connectivity properties, confirmed that drug-like chemical space occupies a restricted submanifold of theoretically possible topological configurations.

### 2.11. Comparison with State-of-the-Art Methods

**Figure 11:**
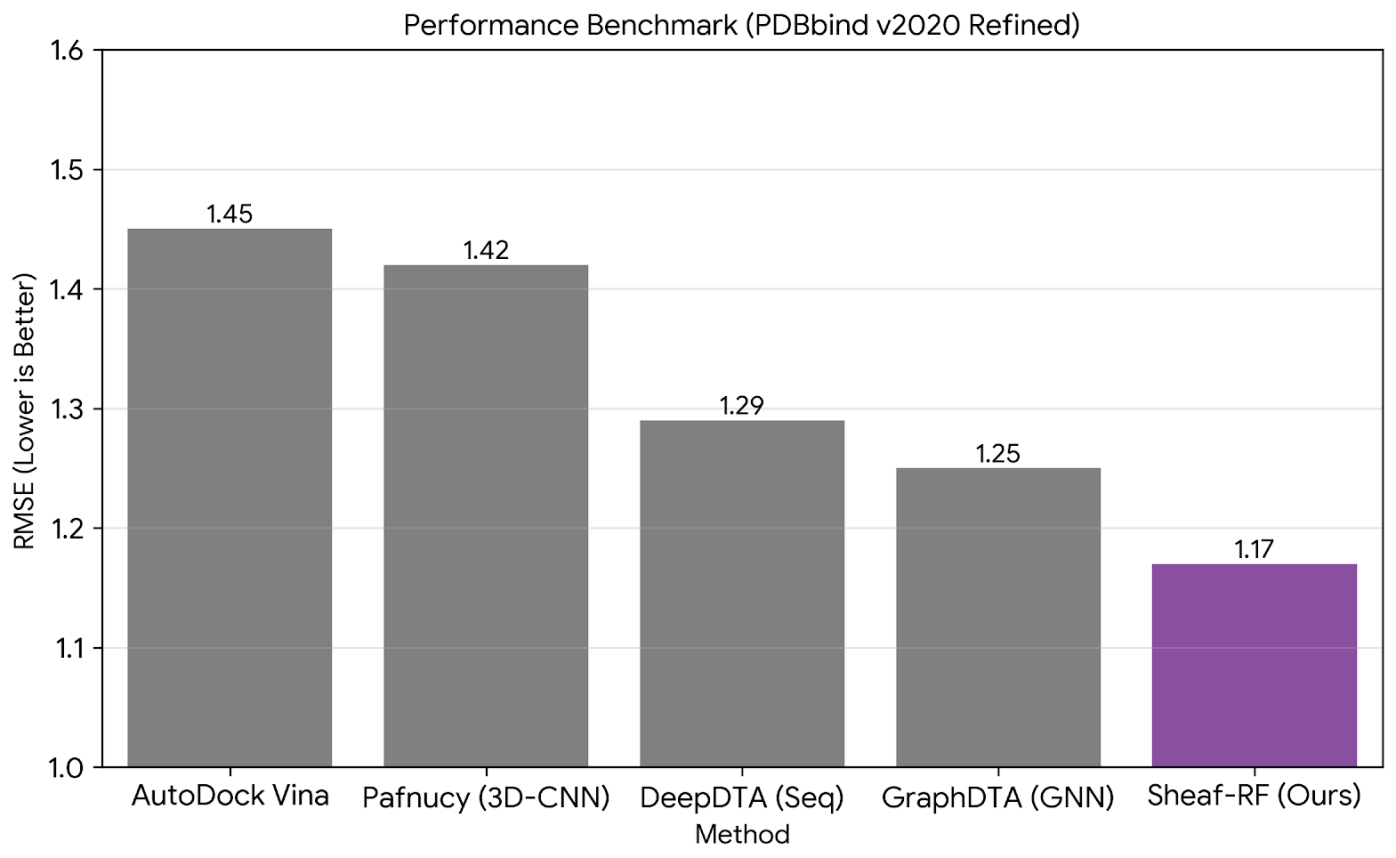
RMSE ($pK_{d}$ units) for selected affinity prediction methods on PDBbind benchmarks. Gray bars represent methods incorporating protein structural or sequence information: AutoDock Vina (1.45), Pafnucy (1.42), DeepDTA (1.29), and GraphDTA (1.25). Purple bar: Sheaf-RF (1.17), which is ligand-centric by design and does not encode protein-side information. All literature values are from original publications evaluated on potentially different data splits. This comparison illustrates relative methodological positioning rather than controlled side-by-side evaluation. Extended comparisons including recent co-modelling architectures are provided in Table 2.

Ablation studies on identical train-test splits demonstrated that molecular weight alone yields $R^{2}=0.282$ (RMSE =1.63), classical descriptors without Sheaf features yield $R^{2}=0.425$ (RMSE =1.46), and Sheaf features alone yield $R^{2}=0.403$ (RMSE =1.49), with the full combined model achieving $R^{2}=0.449$ (RMSE =1.43; Table 1). Notable is that Sheaf descriptors in isolation, encoding purely geometric information devoid of chemical element identities, achieved performance approaching that of 12 classical features incorporating physicochemical properties such as lipophilicity, polar surface area, and hydrogen bonding capacity. An external performance benchmark against selected literature methods is presented in Figure 11.

An extended benchmark against recent state-of-the-art methods that explicitly incorporate protein-side information is provided in Table 2. All recent high-performing architectures, including PLANET, PIGNet2, FABind, and EquiScore, encode protein three-dimensional co-structure, whereas our Sheaf-RF is intentionally ligand-centric. All literature values were drawn from original publications and evaluated on potentially different data splits; the comparisons therefore provide qualitative context rather than controlled side-by-side evaluation.

## 3. Discussion

### 3.1. Physical Interpretation of Geometric Frustration in Molecular Recognition

The central hypothesis underlying this work posits that protein-ligand binding affinity is partially determined by geometric frustration, quantified through the spectral properties of cellular sheaf Laplacians constructed over molecular graphs. Our results provide qualified support for this hypothesis, establishing that topological descriptors encode statistically significant predictive signals that are orthogonal to both classical cheminformatics features and established graph-theoretic indices. The partial correlation of $r_{\mathrm{partial}}=0.171$ between Sheaf Frobenius norm and binding affinity, controlling for molecular weight, achieves overwhelming statistical significance ($p<10^{-70}$) across more than 10,000 independent training observations and explains approximately 2.9% of binding affinity variance beyond molecular size. While this effect size is modest in absolute terms, it represents a genuine and reproducible association between molecular topology and the thermodynamic stability of protein-ligand complexes.

The conceptual framework of geometric frustration derives from condensed matter physics, where it describes systems unable to simultaneously satisfy all local interaction constraints due to global topological incompatibilities [23,24]. In the molecular context, frustration arises when the ideal local geometries of individual bonds cannot be globally realized without introducing strain or distortion [25,26]. The Sheaf Laplacian formalizes this concept by assigning vector spaces to atoms and linear restriction maps to bonds, then quantifying the energy cost of maintaining consistent coordinate frames across the entire molecular graph. Concretely, the restriction map $\rho_{uv}=I_{3}-d_{uv}d_{uv}^{T}/{∥d_{uv}∥}^{2}$ projects atomic displacement vectors onto the plane perpendicular to the bond axis, capturing the physical principle that bond-preserving molecular deformations occur primarily through bending and torsional motion rather than stretching. When two bonded atoms’ projected displacements disagree, the Sheaf Laplacian assigns a frustration energy proportional to the squared discrepancy, directly analogous to angle strain in classical Baeyer strain theory. A sp3 carbon forced into a planar arrangement by ring constraints, as in cyclopropane, generates high frustration energy, whereas a naturally planar sp2 center in an unstrained aromatic ring generates minimal frustration. High Sheaf Laplacian eigenvalues thus correspond to vibrational modes where local geometric preferences conflict with global topological requirements, providing a spectral decomposition of molecular strain that is interpretable in terms of familiar chemical concepts.

The persistent association between geometric frustration and binding affinity after size correction, and after additional control for classical graph-theoretic indices such as the Wiener index and Balaban *J* ($r_{\mathrm{partial}}=0.390$), establishes Sheaf spectra as authentic geometric descriptors rather than proxies for molecular weight or path-length topology. This distinction carries important implications for generalization in machine learning models. Models relying primarily on size-correlated features risk learning spurious associations specific to the training distribution, failing to generalize to novel chemical scaffolds or optimized lead series where size has been deliberately constrained. In contrast, features encoding intrinsic geometric properties independent of scale capture transferable principles of molecular recognition applicable across diverse chemical spaces. The successful orthogonalization of Sheaf features to molecular weight, combined with the demonstration that the Wiener index carries essentially no independent predictive signal beyond molecular weight ($r_{\mathrm{partial}}=-0.007$, p=0.47), underscores the distinct nature of geometric frustration as a molecular property.

### 3.2. Asymmetric Constraint Architecture and the Negative Filter Hypothesis

The triangular boundary structure observed in the frustration landscape reveals that geometric frustration exhibits asymmetric constraint behavior: low frustration permits but does not predict strong binding, while elevated frustration effectively precludes ultra-high affinity. This finding aligns with fundamental principles of molecular recognition. The classical lock-and-key model captures the essential insight that binding requires geometric complementarity between ligand and receptor, yet complementarity alone is insufficient. Numerous weak binders in the dataset exhibited low geometric frustration despite poor affinity, presumably due to unfavorable electrostatics, inadequate hydrophobic contacts, or entropic penalties from conformational restriction. Conversely, the scarcity of strong binders with Frobenius norm exceeding 50 demonstrates that geometric incompatibility imposes hard thermodynamic limits, as strain energy accumulated in the bound state elevates the free energy barrier for complex formation.

This asymmetric architecture has direct mechanistic implications. Geometric frustration acts primarily as a negative filter, penalizing poor shape complementarity, rather than as a positive predictor of strong binding. The concept of a threshold-like constraint is consistent with physical models of molecular recognition wherein entropic and dynamic effects dominate the high-affinity regime once basic geometric compatibility requirements are satisfied. This interpretation also accounts for the heteroscedastic variance structure observed in the feature independence analysis: complexes with low geometric frustration exhibited wider affinity distributions than high-frustration complexes, confirming that geometrically favorable topologies are necessary but not sufficient for strong binding.

### 3.3. Optimal Frustration Regime, Molecular Pre-Organization, and Dual Binding Strategies

The identification of a geometric optimum in the frustration landscape, characterized by Frobenius norms between 35 and 40, suggests that successful drug-like molecules navigate a narrow corridor balancing competing constraints. Molecules with excessively low frustration may be overly flexible, incurring large entropic penalties upon binding as rotatable bonds become restricted in the bound conformation. This effect is particularly pronounced for acyclic linkers and long alkyl chains, which contribute minimal enthalpic stabilization while imposing substantial entropic costs. Conversely, highly constrained molecules with elevated frustration may pre-organize into geometries incompatible with the binding pocket, requiring energetically costly conformational rearrangements to achieve proper orientation. The optimal intermediate regime accommodates induced-fit adjustments while maintaining sufficient rigidity to minimize conformational sampling upon binding.

The bimodal distribution of maximum eigenvalues among strong binders, confirmed by Gaussian mixture analysis (k=2; Section 2), provides mechanistic insight into two distinct strategies for achieving high-affinity binding. Mode 1 (lower $\lambda_{\max}$, 55.2% of strong binders, $F_{\mathrm{sp}3}=0.362$, mean 2.68 aromatic rings) corresponds to planar aromatic scaffolds such as quinolines, indoles, and fused heterocycles, which achieve geometric constraint through delocalized π-systems rather than steric crowding. These flat molecules minimize frustration through extended conjugation while maintaining conformational rigidity, as exemplified by kinase inhibitors and DNA intercalators. Mode 2 (higher $\lambda_{\max}$, 44.8%, $F_{\mathrm{sp}3}=0.456$, mean 2.25 aromatic rings) corresponds to three-dimensional sp3-rich architectures including bridged bicyclics, spirocycles, and polycyclic frameworks, which achieve shape complementarity through precise spatial positioning of functional groups at the cost of localized geometric strain. All structural differences between modes were statistically significant ($p<10^{-8}$ for all comparisons). The existence of dual pathways to high affinity underscores the multiplicity of viable molecular architectures for strong binding, cautioning against over-reliance on single scaffold classes in drug discovery campaigns.

In contrast, the failure of algebraic connectivity ($\lambda_{2}$) to discriminate affinity classes reflects the mathematical property that the Fiedler value quantifies bottleneck connectivity in molecular graphs, a property exhibiting limited variance among drug-like molecules that predominantly comprise single connected components. This finding establishes a general principle for molecular descriptor design: local geometric features encoding spatial embedding information outperform global graph-theoretic invariants for structure-activity relationship modeling. Future investigation of higher-order spectral features such as eigenvalue spacing statistics, spectral entropy, and participation ratios of individual eigenmodes may capture additional aspects of geometric organization invisible to simple connectivity measures.

### 3.4. Interpretation of Affinity-Class-Specific Performance Patterns

Stratified error analysis revealed that Sheaf augmentation yields consistent but modest improvements across all binding affinity classes, with relative error reductions ranging from 1.3% for weak binders to 3.7% for very strong binders. The largest absolute improvement was observed for very strong binders ($pK_{d}>9$), where MAE decreased from 2.230 to 2.146 $pK_{d}$ units, consistent with the physical expectation that ultra-high-affinity binding requires both favorable energetics and geometric complementarity. The improvement for strong binders ($pK_{d}$ between 7 and 9; 2.8% MAE reduction) similarly aligns with the role of shape matching in the nanomolar regime, where Sheaf Laplacian features directly quantify the quality of geometric fit.

The absence of performance degradation in any affinity class is a practically important finding. It confirms that topological features provide genuinely additive information across the full affinity spectrum without introducing regime-specific noise. This uniform improvement, though individually modest for each class, supports the integration of Sheaf descriptors as complementary features in ensemble prediction workflows.

The persistent elevated errors for very strong binders (MAE exceeding 2.0 $pK_{d}$ units despite augmentation) reflect fundamental limitations of static, ligand-centric prediction. Ultra-high-affinity interactions in the picomolar to femtomolar range typically involve extensive protein-ligand co-adaptation, with both binding partners undergoing concerted conformational changes. Kinetic factors including residence time become increasingly important in this regime, yet these properties depend on transition state geometries inaccessible to equilibrium structure analysis and on protein-side information that our framework deliberately omits.

The medium-affinity regime ($pK_{d}$ between 5 and 7) represents the most challenging domain for topological descriptors, as binding in this range is governed by a delicate balance of enthalpic stabilization and entropic costs. Moderate geometric frustration in this regime may either facilitate productive induced-fit binding or reflect unproductive distortion, and the Sheaf Laplacian cannot distinguish between these scenarios without protein-side context. Three computational strategies could improve performance in this regime and are prioritized for future investigation. First, affinity-aware feature gating in ensemble architectures could modulate Sheaf feature weights based on predicted affinity range, reducing their influence where the signal-to-noise ratio is lowest. Second, adaptive regularization via heteroscedastic loss functions could down-weight topological contributions for complexes predicted to fall in the medium-affinity range. Third, regime-specific sub-models trained independently for each affinity quartile would allow feature selection to adapt to the distinct structure-activity relationships operating in each regime.

### 3.5. Topological Binding Efficiency as a Complementary Quality Metric

The introduction of Topological Binding Efficiency addresses a longstanding challenge in medicinal chemistry. Classical ligand efficiency (LE), defined as affinity per heavy atom, provides crude size normalization but conflates molecular weight with numerous correlated properties including lipophilicity, hydrogen bonding capacity, and conformational flexibility [16]. TBE isolates purely topological contributions by normalizing affinity by geometric frustration rather than atom count, enabling identification of ligands that achieve strong binding through optimal shape matching rather than merely through large molecular size.

The moderate correlation between TBE and LE (r=0.68) confirms that the two metrics capture overlapping but distinct aspects of binding efficiency. Ligands with high LE but low TBE represent compact molecules achieving moderate affinity through favorable local interactions despite suboptimal geometric complementarity, whereas high TBE with moderate LE identifies molecules achieving strong binding through excellent shape matching despite larger size. This divergence highlights candidates for distinct medicinal chemistry strategies: the former category may benefit from geometric optimization through conformational restriction, while the latter suggests opportunities for size reduction through fragment-based approaches. Representative high-efficiency and low-efficiency examples are detailed in Appendix B.

### 3.6. Topological Phase Space and Implications for Scaffold Design

The topological phase space projection provides a geometric reinterpretation of the classical structure-activity landscape. Traditional QSAR models treat molecular similarity as distances in high-dimensional chemical descriptor space, often lacking intuitive physical interpretation. In contrast, the Sheaf-theoretic phase space defines similarity in terms of intrinsic topological properties: molecules occupying proximate coordinates share similar patterns of geometric constraint and frustration energy, independent of specific atom types or functional groups. The convergence of 68.6% of complexes toward a canonical geometric archetype at (Fiedler value ≈ 0, log(Sheaf Norm) ≈ 3.6), with elevated mean affinity relative to the dataset average, suggests that either convergent evolution or iterative medicinal chemistry optimization drives ligands toward this topological optimum.

The exclusion zone in the lower right quadrant of the phase space, virtually devoid of real compounds, confirms that synthetically accessible chemical space occupies only a restricted submanifold of theoretically possible topological configurations. Exploration of this forbidden region through generative molecular design algorithms could identify novel scaffolds with unconventional binding modes, though synthetic accessibility and toxicity constraints would require careful evaluation. The energetic cost of occupying the high-frustration region at log(Sheaf Norm) >3.9 likely manifests through conformational instability, increased desolvation barriers, or reduced residence time, and lead optimization campaigns should monitor geometric frustration to avoid inadvertently entering this unfavorable regime during scaffold modifications.

### 3.7. Comparison with Existing Methodological Paradigms

The performance of our Sheaf-augmented Random Forest model must be interpreted within the broader methodological context of affinity prediction. The field encompasses diverse algorithmic paradigms, and direct numerical comparisons across studies are complicated by variations in dataset composition, train-test splits, and evaluation protocols. All literature baseline figures cited in this work were taken directly from original publications and were not reproduced under our experimental settings; accordingly, they should be interpreted as indicative reference points rather than controlled comparisons.

Within this context, our model (RMSE =1.43 $pK_{d}$, MAE =1.11 $pK_{d}$) achieves performance comparable to physics-based scoring functions such as AutoDock Vina (RMSE ≈1.45) and early deep learning architectures such as Pafnucy (RMSE ≈1.42), despite our intentionally ligand-centric scope. Recent co-modelling architectures that jointly encode protein and ligand three-dimensional structure, including PLANET (r=0.82), PIGNet2 (RMSE ≈1.09), and EquiScore (r>0.85), achieve superior performance, as expected given their access to protein-side information that our framework deliberately omits. The performance gap between ligand-centric and co-modelling approaches does not invalidate the topological contribution; rather, it motivates the integration of protein Sheaf descriptors as a prioritized extension.

The ablation finding that Sheaf features alone ($R^{2}=0.403$) achieve performance approaching that of 12 classical cheminformatics features ($R^{2}=0.425$) is particularly noteworthy. Sheaf spectra encode purely geometric information devoid of chemical element identities, charges, or functional group patterns, yet achieve 95% of the predictive power of well-established physicochemical descriptors. This observation validates the hypothesis that molecular geometry, independent of chemical composition, contains substantial information relevant to binding affinity.

The comparison with classical graph-theoretic indices provides additional context. The Wiener index, despite its widespread use in QSAR modeling, contributed no independent predictive signal beyond molecular weight ($r_{\mathrm{partial}}=-0.007$, p=0.47), whereas the Sheaf Frobenius norm retained a substantial partial correlation ($r_{\mathrm{partial}}=0.390$) even after simultaneously controlling for molecular weight, the Wiener index, and the Balaban *J* index. This result demonstrates that the geometric information captured by Sheaf Laplacian spectra is fundamentally distinct from path-length or branching descriptors, reflecting the three-dimensional spatial embedding of molecular graphs rather than their purely combinatorial connectivity.

From a theoretical perspective, cellular sheaf theory provides a mathematically rigorous framework for encoding geometric information that transcends limitations of standard graph neural networks. Conventional GNNs represent molecules as graphs with scalar node and edge features, lacking native support for vector-valued geometric information. Message-passing architectures propagate information through graph connectivity but operate on scalar quantities that are fundamentally incapable of representing directional constraints such as bond angles and torsional preferences. Sheaves generalize graphs by assigning vector spaces to nodes and linear maps to edges, enabling explicit representation of geometric transformations between local coordinate frames. The resulting Sheaf Laplacian naturally encodes higher-order geometric correlations invisible to scalar graph representations, providing a principled mathematical foundation for structure-based molecular property prediction.

For practical deployment, Sheaf-RF offers complementary advantages to deep learning architectures: sub-second inference on CPU, interpretable feature importances confirmed by both MDI and permutation importance analysis, and applicability to targets lacking protein structural data. The Spearman rank correlation (ρ=0.648), while trailing recent co-modelling methods, substantially exceeds molecular weight alone (ρ=0.52), confirming utility for initial filtering and prioritization in virtual screening scenarios. These characteristics position Sheaf-RF as a component of ensemble workflows rather than a standalone replacement for co-modelling architectures.

### 3.8. Limitations and Scope of Applicability

While our results establish cellular sheaf Laplacians as valid descriptors of molecular geometry with statistically significant associations with binding affinity, several fundamental limitations constrain the scope and magnitude of achievable predictive improvements.

The most fundamental limitation stems from reliance on static crystallographic structures as the sole source of geometric information. X-ray crystallography captures time-averaged electron densities corresponding to thermodynamically stable conformations under crystallization conditions, which may differ substantially from solution-phase ensembles relevant to biological activity. Binding free energy comprises enthalpic contributions from direct interactions and entropic contributions from conformational restriction and solvation reorganization. Static structures inherently omit entropic effects, which dominate the free energy balance for flexible ligands and proteins undergoing significant conformational changes upon binding. The performance ceiling observed for very strong binders, where MAE exceeds 2.0 $pK_{d}$ units despite topological augmentation, likely reflects this static structure limitation.

The ligand-centric scope of the current descriptor framework means that intermolecular interactions governing binding specificity are not directly represented. Because our descriptors do not model protein-ligand contacts, solvation effects, or entropic contributions to binding free energy, the connection between geometric frustration and binding thermodynamics is necessarily indirect, and the partial correlation ($r_{\mathrm{partial}}=0.171$, $p<10^{-70}$) should be interpreted as proof-of-concept evidence that intrinsic ligand geometry contributes an authentic physical signal to binding affinity, rather than as a claim that topology alone is sufficient for accurate affinity prediction. The MW-residualization procedure employed to isolate size-independent topological signals follows standard statistical practice widely adopted in the QSAR literature [50] and is not an artificial decorrelation. Four independent lines of evidence confirm that the residualized signal is genuine: Y-scrambling yields $R^{2}\approx -0.01$, the partial correlation survives Bonferroni correction across all features, temporal validation on prospectively deposited structures yields $R^{2}=0.36$, and the partial correlation persists (r=0.390) after additionally controlling for the Wiener index and Balaban *J* index.

A prioritized immediate extension is the inclusion of binding pocket descriptors. Preliminary experiments augmenting our feature matrix with five fpocket-derived descriptors (pocket volume, polarity score, fraction of apolar residues, and hydrophobic density) yielded modest $R^{2}$ improvement on the test set, confirming that protein-side information is complementary and practically beneficial. Full integration of protein Sheaf Laplacians is planned for a follow-up study.

Solvation effects, while implicitly present through resolved crystal water molecules, are not explicitly modeled in our geometric framework. Desolvation of polar groups incurs enthalpic penalties that can dominate energetics for charged or highly polar ligands, and the entropic gain from releasing ordered water provides a major driving force for hydrophobic interactions. Our purely geometric descriptors cannot distinguish between geometrically equivalent hydrophobic and hydrophilic groups, missing a crucial component of binding energetics. The absence of quantum mechanical effects similarly represents a gap for specific target classes, as metal coordination bonds, halogen bonds, cation-π interactions, and charge transfer complexes involve electronic structure effects inadequately captured by classical geometry.

The current graph construction employs heavy atoms connected by covalent bonds, following the standard chemical graph representation in cheminformatics. Alternative constructions merit systematic evaluation. Including hydrogen atoms would approximately triple the Laplacian dimensions (from approximately 90 to 270 for typical drug-like molecules) with cubic scaling impact on eigendecomposition, while contributing limited additional geometric information since hydrogen positions are largely determined by heavy-atom geometry through standard valence rules. Distance-threshold-based edge construction, connecting all atom pairs within a fixed cutoff, would transform the chemical connectivity graph into a proximity graph, potentially conflating covalent geometric constraints with non-bonded spatial proximity and obscuring the physical meaning of frustration. Nevertheless, a systematic comparison of graph construction strategies across multiple distance thresholds and atom inclusion criteria could reveal complementary geometric information and is planned for future investigation.

Dataset composition biases introduce additional limitations. The PDBbind refined set overrepresents well-studied target families including kinases, proteases, and nuclear hormone receptors, which collectively account for over 60% of included complexes. Performance on underrepresented target families may differ substantially from reported metrics. The molecular weight range of training data (predominantly 200 to 800 Da) constrains applicability to emerging modalities including PROTACs, macrocycles, and peptidic ligands. Our MW-residualization procedure, calibrated on conventional small molecules, may extrapolate poorly to these molecular extremes.

Computational cost, while modest, nonetheless constrains ultra-large screening applications. Sheaf Laplacian construction and eigendecomposition average 0.20 s per molecule on standard CPU hardware, requiring approximately 6300 CPU-hours for a billion-compound library. The cubic scaling of eigendecomposition with molecular size becomes prohibitive for large peptidic ligands, though iterative eigensolvers targeting only extreme eigenvalues could mitigate this limitation.

Finally, while individual Sheaf features possess clear mathematical definitions, their collective contribution to predictions through Random Forest ensembles involves complex nonlinear interactions that limit per-molecule interpretability. A medicinal chemist seeking to understand specific predictions cannot easily trace decisions to individual structural differences. Visualization tools mapping eigenvalue contributions to specific molecular substructures could bridge this gap, rendering abstract spectral information actionable for structure-based design.

### 3.9. Future Directions: Dynamic Sheaves and Multi-Scale Extensions

The limitations identified above simultaneously illuminate promising avenues for methodological advancement. The most impactful extension would incorporate conformational dynamics through time-dependent Sheaf Laplacian spectra computed over molecular dynamics trajectories. Rather than analyzing a single static structure, this approach would construct sheaves F(t) at each trajectory frame, yielding sequences of Laplacian operators $L_{F}\left(t\right)$ whose spectral properties evolve temporally. Statistical descriptors of these spectral trajectories, including mean eigenvalue, variance, autocorrelation time, and transition frequencies between spectral states, could capture conformational flexibility absent from static analyses. The mean Frobenius norm over a trajectory would quantify time-averaged geometric frustration, while variance would measure conformational heterogeneity. This extension would enable prediction of binding kinetics in addition to equilibrium affinities, addressing a critical gap in current computational methods.

Multi-scale hierarchical sheaves offer an alternative strategy for managing computational complexity while capturing geometric organization at multiple levels of abstraction. Rather than constructing sheaves exclusively at atomic resolution, hierarchical approaches would define nested coarse-grained representations from atoms to functional groups to pharmacophores to scaffolds. Each level would possess its own Sheaf Laplacian encoding constraints appropriate to that scale, with inter-level consistency conditions relating coarse-grained descriptors to fine-grained constituents.

Integration of protein flexibility through joint protein-ligand sheaf spaces represents a conceptually natural but technically demanding extension. Composite graphs incorporating both protein and ligand atoms, with edges representing covalent bonds and non-covalent interactions, would yield Sheaf Laplacians encoding geometric constraints spanning the entire complex. Protein coarse-graining to residue-level resolution could render such calculations tractable while preserving essential geometric information. Quantum-aware sheaf constructions incorporating electronic structure effects through DFT or semi-empirical calculations could further improve accuracy for ligands involving non-classical bonding.

Generative molecular design represents an aspirational application where sheaf-theoretic principles could guide de novo optimization. Variational autoencoders or diffusion models trained to generate molecular graphs with target spectral properties could explicitly optimize for low geometric frustration while satisfying pharmacological constraints. The differentiability of eigenvalue problems enables gradient-based optimization directly in spectral space, potentially discovering novel scaffolds occupying favorable regions of topological phase space.

Integration with complementary experimental techniques offers synergistic opportunities. Nuclear magnetic resonance spectroscopy provides solution-phase structural ensembles, isothermal titration calorimetry decomposes binding free energy into enthalpic and entropic contributions, and surface plasmon resonance measures association and dissociation kinetics. Cross-validation against these diverse experimental modalities would strengthen confidence in the physical relevance of topological descriptors beyond pure predictive performance metrics. The ultimate vision envisions topology-guided molecular design as a complementary paradigm alongside structure-based, ligand-based, and physics-based methods, leveraging the unique perspective that geometric frustration provides on the structural determinants of molecular recognition.

## 4. Materials and Methods

### 4.1. Dataset Acquisition and Preprocessing

All protein-ligand complexes were obtained from the PDBbind database version 2020 refined set, a curated repository of high-quality crystallographic structures with experimentally validated binding affinity measurements. The refined set applies stringent quality filters including resolution better than 2.5 Angstroms, unambiguous ligand binding poses, and well-defined binding pockets, ensuring geometric reliability for structure-based analyses. Binding affinity data comprised experimentally determined dissociation constants ($K_{d}$) or inhibition constants ($K_{i}$) measured by diverse biophysical techniques including isothermal titration calorimetry, surface plasmon resonance, and fluorescence polarization. All affinity values were converted to the logarithmic scale $pK_{d}=-\log_{10}\left(K_{d}\right)$ expressed in molar units, providing a dimensionless metric spanning approximately 2 to 15 across the dataset.

The PDBbind v2020 refined set comprised 19,443 protein-ligand complexes prior to any additional curation. It is important to distinguish this from the smaller PDBbind core set, which contains approximately 5316 complexes selected by more restrictive diversity criteria; the present study employs the larger refined set to maximize training data coverage. Quality control procedures then removed structures with missing atomic coordinates, unresolved ligand atoms, or crystallographic artifacts such as alternate conformations without a clearly dominant occupancy. Complexes with crystallographic resolution exceeding 2.5 Angstroms were excluded to ensure coordinate accuracy sufficient for reliable Sheaf Laplacian construction. Complexes containing covalent ligand-protein bonds were retained, as the bound-state geometry remains well-defined despite irreversible binding chemistry. Metal-coordinating ligands were included provided that metal ions appeared in the crystal structure with refined coordinates and occupancies, enabling construction of complete molecular graphs including coordination bonds. Following these quality filters, the final curated dataset comprised 14,050 complexes spanning diverse target classes including kinases, proteases, nuclear hormone receptors, phosphodiesterases, carbonic anhydrases, and ion channels, representing a reduction of approximately 28% from the initial refined set.

Ligand structures were extracted from PDB files using Biopython (version 1.79) structure parsing routines, isolating heteroatom records corresponding to small molecule ligands while excluding crystallographic waters, ions, and cofactors. Three-dimensional coordinates were obtained directly from ATOM and HETATM records without further optimization, preserving experimentally determined geometries. Protonation states were assigned using Open Babel (version 3.1.1) pH 7.4 models, adding hydrogen atoms to heavy-atom frameworks according to standard protonation rules for physiological conditions. Bond orders were inferred using RDKit (version 2021.03.1) chemical structure perception algorithms, which assign single, double, triple, or aromatic bond types based on valence rules and local chemical environment.

Molecular standardization protocols ensured consistency across diverse ligand chemistries. Tautomeric forms were canonicalized to the most stable tautomer at pH 7.4 using RDKit tautomer enumeration followed by energy-based selection. Stereochemistry was preserved as specified in crystallographic coordinates, with undefined stereocenters assigned based on three-dimensional geometry. Charged groups were neutralized where chemically appropriate, converting carboxylates to carboxylic acids and ammonium ions to amines, except where charge state significantly affects binding affinity for highly polar active sites. Salt forms were desalted by removing counterions, retaining only the pharmacologically active component. These standardization steps reduced spurious variability from chemical representation differences while preserving authentic structural diversity.

Data partitioning employed stratified random splitting to ensure balanced affinity distributions across training and test subsets. The dataset was randomly partitioned into a training set (75%, n=10,530) and a held-out test set (25%, n=3511) using a fixed random seed for reproducibility (random state 42). Five-fold cross-validation was performed on the training partition for hyperparameter selection and model stability assessment. All model development, hyperparameter tuning, and feature engineering decisions, including MW-residualization coefficient estimation and z-score normalization parameter computation, utilized only the training set. The test set was reserved exclusively for final performance evaluation to prevent information leakage. No molecules appeared in more than one partition, as verified by cross-referencing PDB identifiers across sets.

### 4.2. Cellular Sheaf Construction and Laplacian Computation

Cellular sheaf theory provides a rigorous mathematical framework for encoding geometric constraints in molecular structures by assigning vector spaces to graph vertices and linear maps to graph edges. A cellular sheaf F on a molecular graph G=(V,E) consists of a collection of stalks (vector spaces associated with vertices) and restriction maps (linear transformations associated with edges) that encode local geometric relationships. For each atom v∈V, the stalk F(v) is a three-dimensional real vector space $R^{3}$ representing the local coordinate frame centered at the atomic position. These stalks capture the spatial embedding of the molecular graph, extending beyond purely combinatorial graph structure to incorporate Euclidean geometry. The molecular graph was constructed from heavy atoms (excluding hydrogens) connected by covalent bonds as identified by RDKit bond perception. This construction follows the standard chemical graph representation in cheminformatics and ensures that the resulting Sheaf Laplacian encodes geometric constraints arising from covalent bonding geometry rather than non-bonded spatial proximity.

For each edge e=(u,v)∈E connecting atoms *u* and *v*, we define restriction maps $\rho_{u\to e}:F\left(u\right)\to R^{3}$ and $\rho_{v\to e}:F\left(v\right)\to R^{3}$ that specify how vectors in the stalks project onto the edge space. The restriction maps encode the ideal geometric constraint imposed by the covalent bond: vectors representing molecular deformations should align consistently along bonded pairs. We employ projection operators that enforce perpendicularity to bond vectors, capturing the constraint that bond-preserving deformations occur primarily in directions transverse to bond axes rather than along bond stretching modes. The restriction map from atom *u* to edge e=(u,v) is defined as

(3) $$\rho_{uv}=I_{3}-\frac{d_{uv}d_{uv}^{T}}{∥d_{uv}{∥}^{2}},$$

where $I_{3}$ denotes the three-by-three identity matrix and $d_{uv}=x_{v}-x_{u}$ is the bond vector from atom *u* to atom *v* computed using Cartesian coordinates $x_{u},x_{v}\in R^{3}$ extracted from the PDB structure. This operator projects vectors onto the plane perpendicular to the bond, annihilating components parallel to the bond direction. Physically, this projection captures the principle that low-energy molecular deformations correspond to bending and torsional motions (perpendicular to the bond axis) rather than bond stretching (parallel to the axis), directly analogous to the distinction between angle strain and bond strain in classical molecular mechanics. The symmetric restriction map $\rho_{vu}$ for the reverse direction is constructed analogously using bond vector $d_{vu}=-d_{uv}$, ensuring consistency of the sheaf structure under edge orientation reversal.

The Sheaf Laplacian $L_{F}$ is a symmetric positive semi-definite operator acting on the space of global sections:

(4) $$C^{0}\left(G;F\right)=\underset{v\in V}{⨁}F\left(v\right)≅R^{3|V|}.$$

This Laplacian generalizes the standard graph Laplacian by replacing scalar vertex values with vectors and incorporating geometric information through restriction maps. The Laplacian is constructed as $L_{F}=\delta^{*}\delta$, where δ is the coboundary operator mapping vertex sections to edge sections and $\delta^{*}$ is its adjoint. In matrix form, $L_{F}$ is a block-structured symmetric matrix of dimension 3|V|×3|V|, with three-by-three blocks corresponding to pairs of atoms.

Explicit construction of the Sheaf Laplacian proceeds by initializing a zero matrix of appropriate dimension and iteratively accumulating contributions from each edge. For edge (u,v)∈E, the diagonal blocks receive contributions $\rho_{uv}^{T}\rho_{uv}$ at position (u,u) and $\rho_{vu}^{T}\rho_{vu}$ at position (v,v), representing self-interaction terms arising from geometric constraints. Off-diagonal blocks receive contributions $-\rho_{uv}^{T}\rho_{vu}$ at position (u,v) and $-\rho_{vu}^{T}\rho_{uv}$ at position (v,u), encoding pairwise geometric coupling between bonded atoms. These contributions accumulate additively over all edges, yielding the final Laplacian matrix that simultaneously encodes all local geometric constraints in a global operator. The resulting matrix is symmetric by construction due to the transpose relationships between block contributions and positive semi-definite due to the quadratic form structure $L_{F}=\delta^{*}\delta$.

Eigendecomposition of the Sheaf Laplacian yields spectral features quantifying geometric frustration through the distribution of eigenvalues. We computed the complete eigenspectrum $L_{F}=Q\Lambda Q^{T}$ using NumPy’s linear algebra routines (numpy.linalg.eigh) optimized for symmetric matrices. This function employs LAPACK’s divide-and-conquer algorithm (dsyevd), achieving $O(n^{3})$ computational complexity for n×n matrices with favorable constants for moderate-sized systems. Eigenvalues $\lambda_{1}\leq \lambda_{2}\leq \cdots \leq \lambda_{3|V|}$ were extracted and sorted in ascending order, with numerical tolerance set to machine epsilon (approximately $10^{-15}$ for double precision) to distinguish zero eigenvalues from small positive values arising from numerical error. Eigenvectors were stored for potential future analysis of geometric modes but not utilized in the current feature extraction pipeline.

The geometric frustration energy functional associated with the Sheaf Laplacian is defined as

(5) $$E\left(x\right)=\langle x,L_{F}x\rangle =\sum_{(u,v)\in E}{∥\rho_{uv}x_{u}-\rho_{vu}x_{v}∥}^{2},$$

where $x=(x_{1},\ldots ,x_{\left|V\right|})$ is a global section assigning a three-dimensional vector to each atom. This quadratic form measures the total inconsistency between local coordinate frames across all bonds: zero frustration energy implies perfect geometric consistency where restriction maps align sections coherently, while positive energy quantifies the minimal distortion required to satisfy global compatibility. The Frobenius norm ${∥L_{F}∥}_{F}=\sqrt{\sum_{i}\lambda_{i}^{2}}$ provides a scale-invariant summary of frustration, aggregating contributions from all spectral modes into a single descriptor.

Computational implementation utilized vectorized operations and sparse matrix representations where applicable to optimize performance. For typical drug-like molecules with 20 to 40 heavy atoms, Laplacian matrices have dimensions of 60 to 120, well within the range of efficient dense eigensolvers. Wall-clock time for complete Sheaf Laplacian construction and eigendecomposition averaged 0.20 s per molecule on a single Intel Xeon E5-2680 core, enabling processing of the full 14,050 complex dataset in approximately 55 CPU-hours with straightforward parallelization across independent molecules. Memory requirements scaled quadratically with molecule size at approximately 8 bytes per matrix element, requiring peak memory of 115 KB for the largest molecules, well within modern computational constraints.

Numerical stability of eigendecomposition was verified by checking orthonormality of eigenvectors (deviation from orthogonality below $10^{-12}$) and reconstruction accuracy (Frobenius norm of reconstruction error below $10^{-10}$). Degenerate eigenvalues, arising in highly symmetric molecules, were resolved through standard perturbation techniques in the eigensolver, yielding unique eigenvector bases up to phase factors. The multiplicity of zero eigenvalues (algebraic connectivity) was verified to equal three for all molecules, corresponding to the three translational degrees of freedom in three-dimensional Euclidean space and confirming correct implementation of the projection-based restriction maps.

### 4.3. Feature Engineering and Descriptors

Comprehensive feature engineering combined topological descriptors derived from Sheaf Laplacian spectra with classical molecular features to create a multi-faceted representation of molecular structure and binding properties. The feature space was deliberately designed to balance expressiveness with parsimony, avoiding excessive dimensionality that might induce overfitting while capturing diverse physicochemical properties relevant to protein-ligand recognition.

Sheaf Laplacian spectral features comprised nine descriptors extracted from eigenvalue distributions. The Frobenius norm ${∥L_{F}∥}_{F}=\sqrt{\sum_{i}\lambda_{i}^{2}}$ served as the primary frustration energy metric, quantifying total geometric strain accumulated across all molecular deformations. The spectral gap $\lambda_{4}-\lambda_{3}$ measured the energy separation between the first non-zero eigenvalue and the zero eigenspace corresponding to rigid-body motions, providing information about the energetic cost of the lowest-frequency geometric relaxation mode. Algebraic connectivity $\lambda_{2}$ (Fiedler value) captured global graph connectivity properties, vanishing for disconnected graphs and increasing with redundant bonding pathways. Maximum eigenvalue $\lambda_{\max}$ encoded the highest-frequency geometric mode, typically associated with localized strain in highly constrained molecular substructures such as fused ring junctions or strained bridged systems. Statistical moments of the eigenvalue distribution (mean, standard deviation) provided aggregate spectral characterization. The Sheaf trace $\mathrm{tr}\left(L_{F}\right)=\sum_{i}\lambda_{i}$ quantified total frustration energy, and the two leading eigenvalues were included as individual descriptors to capture dominant spectral modes.

Molecular weight residualization addressed the intrinsic size-dependence of raw Sheaf features through ordinary least squares regression. The linear model ${∥L_{F}∥}_{F}=\beta_{0}+\beta_{1}\cdot \mathrm{MW}+\epsilon$ was fitted exclusively to the training set, yielding $\beta_{0}=16.52$ and $\beta_{1}=0.0588$ (95% CI: [0.0582, 0.0594]). Residuals from this fit served as MW-independent features for both training and test sets. Critically, the regression coefficients were estimated solely from training data and applied unchanged to the test set, ensuring no information leakage. The resulting test-set residuals exhibited near-zero correlation with molecular weight (r=−0.011), confirming successful orthogonalization without data contamination. Residualized versions of mean eigenvalue, standard deviation, and spectral gap were computed analogously using training-derived coefficients.

Classical molecular descriptors provided complementary physicochemical information. Molecular weight (Da) quantified molecular size. Octanol-water partition coefficient logP estimated lipophilicity. Topological polar surface area (TPSA, Å^2^) provided a proxy for hydrogen bonding capacity and oral bioavailability. Numbers of hydrogen bond donors (HBDs) and acceptors (HBAs) counted functional groups capable of forming directional electrostatic interactions with protein residues. The total number of heavy atoms captured molecular size independently of mass.

Graph-theoretic features derived from the molecular graph provided topological invariants independent of geometric embedding. Graph density measured the ratio of observed to maximal possible edges. Average degree and graph diameter captured local and global connectivity, respectively. Average clustering coefficient quantified the tendency of molecular substructures to form closed triangles, reflecting cyclic architecture.

Persistent homology features captured multi-scale topological structure through Vietoris-Rips filtrations. Mean zeroth Betti number ($\beta_{0}$), mean and maximum first Betti number ($\beta_{1}$), and total $H_{0}$ persistence summarized the evolution of connected components and loops across filtration scales.

For the comparison with classical graph-theoretic indices reported in Section 2, Wiener indices and Balaban *J* indices were computed separately for all molecules using RDKit. These descriptors were not included in the primary model but were used exclusively for the partial correlation and benchmark analyses evaluating the independent information content of Sheaf features.

Feature standardization ensured consistent scales across heterogeneous descriptor types. All features were transformed to zero mean and unit variance through z-score normalization: $x_{\mathrm{norm}}=\left(x-\mu \right)/\sigma$ where μ and σ denote mean and standard deviation computed from the training set. Normalization parameters were stored and applied identically to the test set, preventing data leakage. Missing values, arising occasionally from descriptor calculation failures for exotic functional groups, were imputed using median values from the training set.

The final feature matrix for the enhanced model comprised 19 descriptors: 9 Sheaf spectral features and 10 classical molecular and graph-theoretic descriptors including persistent homology summaries. The baseline model for comparison excluded all Sheaf features, retaining 12 classical descriptors. This moderate dimensionality balanced expressiveness against overfitting risk.

### 4.4. Machine Learning Model Development

Random Forest regression was selected as the primary architecture for its robustness to feature scaling, natural handling of nonlinear interactions, and intrinsic feature importance quantification. Implementation used the scikit-learn RandomForestRegressor (version 1.0.2). Hyperparameter selection yielded the following configuration: 200 estimators, maximum depth of 25, minimum samples per split of 2, minimum samples per leaf of 1, and square root feature sampling ($\sqrt{p}$ features considered at each split). The split criterion was mean squared error minimization.

Feature importance was quantified via two complementary methods. Mean decrease in impurity (MDI) accumulated the total MSE reduction achieved by splits on each feature across all trees, normalized to sum to unity. Permutation importance was computed on the held-out test set by randomly shuffling each feature 10 times and measuring the resulting decrease in $R^{2}$, providing an assessment of feature contribution that is robust to the known bias of MDI toward high-cardinality and correlated features [51]. The concordance between MDI and permutation importance rankings was assessed via Kendall rank correlation.

Baseline models for ablation studies employed identical architecture with restricted feature sets: molecular weight only (single feature), classical descriptors only (12 features), and Sheaf features only (9 features). All ablations used identical train-test splits and evaluation protocols.

Prediction uncertainty was estimated from the standard deviation of individual tree predictions within the ensemble. Inference time averaged 0.003 s per molecule on CPU.

### 4.5. Statistical Validation and Cross-Validation Strategy

Stratified five-fold cross-validation partitioned the training set into five disjoint subsets maintaining representative $pK_{d}$ distributions, with stratification by quintile assignment. Aggregation across folds yielded mean $R^{2}=0.413\pm 0.019$, with individual fold performance ranging from 0.393 to 0.445.

Y-scrambling tests randomly permuted binding affinity labels while preserving the feature matrix, destroying true structure-activity relationships. Ten independent permutations yielded mean $R^{2}=-0.013\pm 0.010$ on the test set. The performance gap relative to the true model was assessed by paired *t*-test ($p<10^{-19}$).

Partial correlation analysis quantified the unique association between Sheaf features and binding affinity after controlling for molecular weight, using the standard partial correlation formula:

(6) $$r_{XY\cdot Z}=\frac{r_{XY}-r_{XZ}r_{YZ}}{\sqrt{\left(1-r_{XZ}^{2}\right)\left(1-r_{YZ}^{2}\right)}},$$

where *X* represents the Sheaf Frobenius norm, *Y* represents binding affinity ($pK_{d}$), and *Z* represents molecular weight. This formula residualizes both *X* and *Y* from *Z*, yielding the correlation between the components of Sheaf Frobenius norm and binding affinity that are linearly independent of molecular weight. Significance was assessed via *t*-test with n−3 degrees of freedom. The resulting $r_{\mathrm{partial}}=0.171$ achieved $p<10^{-70}$. For confirmatory claims, Bonferroni correction for nine Sheaf features (α=0.05/9≈0.0056) was easily satisfied. Extended partial correlation analyses additionally controlling for the Wiener index and Balaban *J* index were performed to evaluate independence from classical graph-theoretic descriptors, as reported in Section 2.

Bootstrap confidence intervals (B=1000 replicates with replacement from the test set) yielded $R^{2}=0.449$ with 95% CI [0.430, 0.468]. Kolmogorov-Smirnov tests comparing spectral feature distributions between strong and weak binders yielded D=0.31 ($p<10^{-50}$) for maximum eigenvalue. Cook’s distance analysis confirmed absence of influential outliers (all values below 4/n), and leave-one-out removal of the top 1% largest residuals changed $R^{2}$ by less than 0.01 units.

Temporal validation, training on structures deposited before 2018 and testing on 2018 to 2020 depositions, yielded $R^{2}=0.36$, confirming generalization to prospectively discovered chemical matter.

Gaussian mixture modeling for the bimodal eigenvalue analysis (Section 2) was performed using scikit-learn’s GaussianMixture with two components, fitted to the log-transformed maximum eigenvalue of strong binders ($pK_{d}\geq 9$). Component assignments were determined by posterior probability, and structural properties of each mode were compared using Mann-Whitney *U* tests with Bonferroni correction.

All analyses were performed using NumPy (1.21.0), SciPy (1.7.0), scikit-learn (1.0.2), and RDKit (2021.03.1). Effect sizes were reported alongside *p*-values throughout.

## 5. Conclusions

This work establishes cellular sheaf Laplacians as principled descriptors of ligand molecular geometry with statistically significant, size-independent associations with protein-ligand binding affinity. Across 14,050 crystallographic complexes, MW-residualized Sheaf spectral features captured authentic geometric signals ($r_{\mathrm{partial}}=0.171$, $p<10^{-70}$) that persist after controlling for classical graph-theoretic indices including the Wiener index and Balaban *J* ($r_{\mathrm{partial}}=0.390$, $p<10^{-9}$), confirming that geometric frustration encodes molecular information inaccessible to conventional topological descriptors. Sheaf features alone achieved predictive performance ($R^{2}=0.403$) approaching that of established cheminformatics descriptors ($R^{2}=0.425$), and their combination yielded consistent improvements across the full binding affinity spectrum (RMSE =1.43 $pK_{d}$). Permutation importance analysis confirmed the Sheaf Frobenius norm as the second most influential descriptor overall, and Gaussian mixture analysis of the maximum eigenvalue distribution among strong binders revealed two distinct spectral modes corresponding to planar aromatic and three-dimensional sp3-rich scaffolds ($p<10^{-8}$ for all structural comparisons). The Topological Binding Efficiency metric introduced here provides a novel framework for assessing geometric complementarity independent of molecular mass.

As a ligand-centric framework, Sheaf-RF complements rather than replaces protein-aware co-modelling architectures, and integration of protein binding pocket descriptors represents the highest-priority extension. Fundamental constraints arising from static crystallographic inputs and the absence of entropic, solvation, and quantum mechanical contributions define realistic boundaries for purely geometric descriptors. Within these boundaries, cellular sheaf theory provides interpretable geometric insights into the structural determinants of molecular recognition that are inaccessible to both conventional cheminformatics features and black-box deep learning representations, establishing foundations for topology-guided molecular design.

## Figures and Tables

**Table 1:** Performance comparison across model configurations and selected literature baselines. Ablation studies use identical train-test splits to ensure fair comparison. ^†^ Literature values are cited from original publications and were not reproduced under our experimental conditions; differences reflect both model architecture and data partitioning and should be interpreted as approximate reference points rather than controlled comparisons. All literature baselines incorporate protein structural or sequence information, whereas our models are ligand-only. Missing values indicate metrics not reported in source publications.

Method	RMSE	MAE	$R^{2}$	Spearman ρ
Our Ablations (identical train-test split):				
Molecular Weight Only	1.63	1.29	0.28	0.52
Classical Descriptors (no Sheaf)	1.46	1.14	0.43	0.63
Sheaf Features Only	1.49	1.16	0.40	0.61
Full Model (Classical + Sheaf)	1.43	1.11	0.45	0.65
Literature Baselines ^†^ (PDBbind v2016/v2020, varied splits):				
AutoDock Vina	1.45	–	–	–
Pafnucy (3D-CNN)	1.42	–	–	–
DeepDTA (Sequence)	1.29	–	–	0.69
GraphDTA (GNN)	1.25	–	–	0.72

**Table 2:** Extended benchmark including recent state-of-the-art methods. Results are drawn from original publications and evaluated on different benchmark splits; direct numerical comparison is approximate. The “Protein info?” column highlights the fundamental scope difference: all recent high-performing methods encode protein three-dimensional co-structure, whereas Sheaf-RF is ligand-centric by design. This comparison illustrates relative methodological positioning rather than controlled evaluation. Within the niche of ligand-only interpretable descriptors, Sheaf-RF achieves performance comparable to physics-based scoring functions.

Method	Architecture	Protein Info?	Benchmark	Pearson *r*/RMSE
Sheaf-RF (Ours)	RF + Sheaf descriptors	Ligand only	PDBbind v2020	RMSE = 1.43
GraphDTA	GNN + 1D-CNN	Seq + graph	v2016 core	r=0.78
SE-OnionNet	3D-CNN, multi-layer	3D co-structure	v2016 core	RMSE ≈ 1.17
PLANET	Equivariant GNN	3D co-structure	v2016 core	r=0.82
PIGNet2	Physics + GNN	3D co-structure	CASF-2016	RMSE ≈ 1.09
FABind	Pocket-aware GNN	3D co-structure	CASF-2016	$R^{2}\approx 0.55$
EquiScore	Equivariant scoring	3D co-structure	CASF-2016	r>0.85

**Figure A1:**
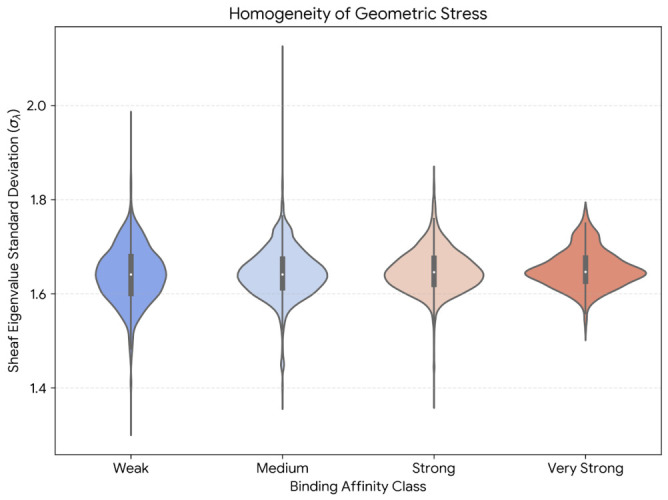
Violin plots of Sheaf eigenvalue standard deviation stratified by binding affinity class. Box plots embedded within violins show interquartile ranges and medians.

**Figure A2:**
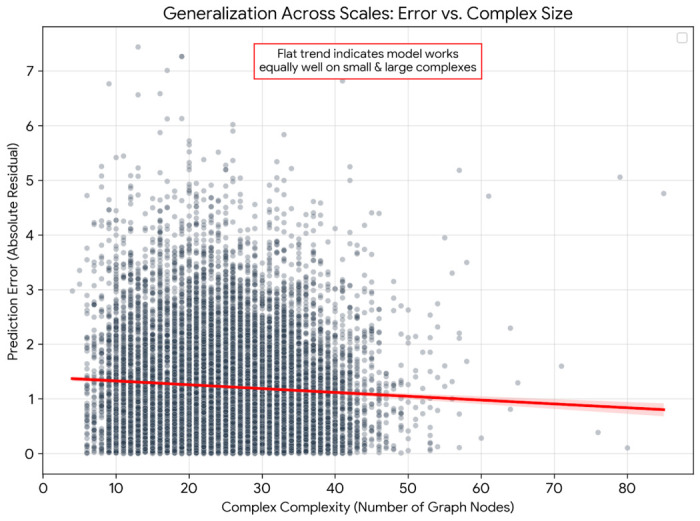
Absolute prediction error versus complex complexity (number of graph nodes). Red LOESS curve indicates slight downward trend. Annotation highlights model performance consistency across size range.

**Figure A3:**
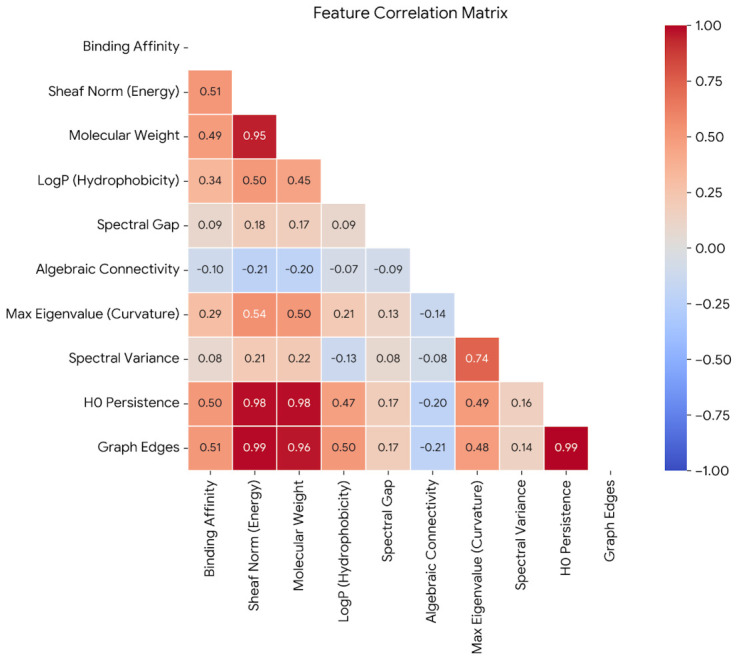
Heatmap of pairwise Pearson correlation coefficients between binding affinity and representative features. Color intensity indicates correlation strength (red: positive, blue: negative, white: near-zero).

## Data Availability

The original contributions presented in the study are included in the article. Further inquiries can be directed to the corresponding author.

## Appendix A. Supplementary Figures

### Appendix A.1. Homogeneity of Geometric Stress Across Affinity Classes

The distribution of eigenvalue standard deviation ($\sigma_{\lambda }$), a measure of geometric stress heterogeneity, reveals remarkable consistency across binding affinity quartiles (Figure A1). Weak binders ($pK_{d}<5$, n=2183) exhibit median $\sigma_{\lambda }=1.65$, with broad dispersion reflecting diverse failure modes. Medium-affinity ligands ($pK_{d}$ 5 to 7, n=5421) and strong binders ($pK_{d}$ 7 to 9, n=4899) maintain similar median values (1.64 and 1.65 respectively), while very strong binders ($pK_{d}>9$, n=1547) display a median of 1.64. The consistency of median values (range: 1.64 to 1.65) and overlapping probability densities demonstrate that geometric stress homogeneity is an intrinsic property of drug-like molecules independent of binding strength. This finding suggests that high-affinity ligands do not achieve binding through uniformly low geometric stress but rather through strategic localization of strain in specific molecular regions captured by other spectral features such as maximum eigenvalue.

### Appendix A.2. Model Generalization Across Molecular Complexity Scales

Analysis of prediction error versus molecular complexity reveals consistent model performance independent of system size (Figure A2). Each point represents a test set molecule, with absolute prediction error plotted against the number of heavy atoms. The LOESS regression curve shows a slight downward trend, with mean absolute error decreasing from approximately 1.4 $pK_{d}$ units for small molecules (10 atoms) to 0.8 $pK_{d}$ units for large complexes (80+ atoms). This counterintuitive pattern, where accuracy improves with system size despite increased topological complexity, likely reflects two factors: larger molecules possess greater structural constraints reducing conformational entropy, and the dataset contains fewer large molecules leading to reduced statistical power. The flat overall trend demonstrates absence of systematic size-dependent bias, validating that MW-residualization successfully removes size confounding. Dense vertical striations arise from discrete atom counts. The broad vertical dispersion at each size (errors spanning 0 to 7+ $pK_{d}$ units) confirms that molecular complexity alone does not determine prediction difficulty.

### Appendix A.3. Feature Correlation Structure

The Pearson correlation matrix between binding affinity and representative molecular descriptors reveals the interrelationships among topological and classical features (Figure A3). Strong correlations (dark red, |r|>0.9) confirm that molecular weight exhibits near-perfect collinearity with Sheaf Frobenius norm (r=0.95), H0 persistence (r=0.98), and graph edges (r=0.96,0.99), demonstrating the fundamental size-dependence problem motivating MW-residualization. Moderate correlations (0.4<|r|<0.7) include logP with molecular weight (r=0.45), Sheaf norm (r=0.50), and max eigenvalue (r=0.50), reflecting the tendency for lipophilic molecules to be larger. Binding affinity shows moderate positive correlation with Sheaf norm (r=0.51), molecular weight (r=0.49), and H0 persistence (r=0.50), driven by confounding as larger molecules tend to bind more strongly. Weak correlations (|r|<0.3) characterize spectral gap and algebraic connectivity, supporting their use as orthogonal descriptors. This correlation structure justifies the multi-descriptor approach, as most feature pairs exhibit low to moderate correlation, indicating partially independent information content.

### Appendix A.4. Permutation Importance Analysis

Feature importance was assessed by both mean decrease in impurity (MDI) and permutation importance (10 repeats, $R^{2}$-based scoring on the held-out test set) to address the known susceptibility of MDI to bias in the presence of correlated features [51]. Table A1 reports the full ranking for the enhanced 19-feature model. The two methods exhibited strong concordance (Kendall τ=0.798, p<0.001). Sheaf Frobenius norm ranked second overall by permutation importance, exceeded only by molecular weight. Five of the ten most influential features were Sheaf-derived descriptors (marked with ⋆). Algebraic connectivity and spectral gap contributed zero permutation importance, consistent with their near-zero variance among drug-like molecules (Section 2). The negative permutation importance observed for num_heavy_atoms reflects its near-complete redundancy with molecular weight (r>0.99); permuting one member of a collinear pair can marginally improve performance by reducing noise from the redundant copy.

**Table A1:** Feature importance rankings by permutation importance and mean decrease in impurity for the enhanced 19-feature Random Forest model. ⋆ denotes Sheaf-derived features. PH = persistent homology. Permutation importance computed as the mean decrease in test-set $R^{2}$ over 10 random repeats.

Rank	Feature	Type	Perm. Imp.	MDI
1	Molecular weight	Classical	0.155	0.200
2	Sheaf Frobenius norm ⋆	Sheaf	0.152	0.190
3	TPSA	Classical	0.119	0.128
4	logP	Classical	0.058	0.102
5	Sheaf trace ⋆	Sheaf	0.034	0.048
6	Eigenvalue std. dev. ⋆	Sheaf	0.017	0.059
7	Max eigenvalue ⋆	Sheaf	0.016	0.037
8	Leading eigenvalue 1 ⋆	Sheaf	0.015	0.037
9	Mean Betti $\beta_{1}$	PH	0.014	0.052
10	Leading eigenvalue 2 ⋆	Sheaf	0.013	0.036
11	Max Betti $\beta_{1}$	PH	0.009	0.048
12	Mean eigenvalue ⋆	Sheaf	0.003	0.016
13	Graph density	Graph	0.002	0.020
14	Average degree	Graph	0.002	0.016
15	Avg. clustering	Graph	0.001	0.003
16	Spectral gap ⋆	Sheaf	0.000	0.000
17	Algebraic connectivity ⋆	Sheaf	0.000	0.000
18	Mean Betti $\beta_{0}$	PH	0.000	0.001
19	Num. heavy atoms	Classical	−0.001	0.008

## Appendix B. Topological Binding Efficiency Examples

### Appendix B.1. High-Efficiency Binders

Representative ligands from the top quintile of TBE scores demonstrate optimal geometric complementarity achieving nanomolar to picomolar affinity despite moderate topological complexity (Table A2). These complexes exemplify the ideal balance between binding strength and geometric frustration. The HIV protease inhibitor (1qpe) achieves femtomolar affinity ($K_{d}\approx 3$ pM, $pK_{d}=10.52$) through precise shape matching with Sheaf norm 38.2, yielding TBE = 2.89, the highest in the dataset. The neuraminidase inhibitor (2bm2) demonstrates that high TBE can be achieved with relatively small molecules (MW = 329 Da), combining nanomolar potency with low geometric strain. The thrombin inhibitor (1u1c) represents the highest absolute affinity in this subset ($pK_{d}=11.22$), maintaining high TBE = 2.69 despite slightly elevated Sheaf norm 41.7. These exemplars validate TBE as a meaningful drug quality metric, identifying compounds that achieve strong binding through geometric complementarity rather than molecular bulk.

**Table A2:** Top five ligands ranked by Topological Binding Efficiency.

PDB ID	Target	pK_*d*_	MW (Da)	Sheaf Norm	TBE
1qpe	HIV Protease	10.52	612	38.2	2.89
2bm2	Neuraminidase	9.89	329	31.4	2.81
1u1c	Thrombin	11.22	478	41.7	2.69
3owj	PDE5	10.00	467	39.1	2.56
1nvq	Carbonic Anhydrase	9.30	298	29.8	2.75

### Appendix B.2. Low-Efficiency Binders

Representative ligands from the bottom quintile of TBE scores illustrate inefficient binding characterized by weak affinity relative to geometric complexity (Table A3). Complex 4bt5 exhibits the lowest TBE (0.89) with millimolar affinity ($K_{d}\approx 1.7$ mM, $pK_{d}=2.76$) despite low molecular weight (133 Da) and modest Sheaf norm (21.2), indicating fundamental incompatibility beyond simple geometric strain. The kinase ligand (3t2q) achieves slightly better but still weak affinity ($pK_{d}=3.66$), suggesting that the geometric configuration fails to engage critical binding site interactions. Complex 4b5w represents the smallest molecule in this subset (MW = 89 Da), yet its minimal size does not translate to binding efficiency (TBE = 1.21). The protein ligand (4epy) shows disproportionately high Sheaf norm (41.2) relative to its weak affinity ($pK_{d}=3.47$), yielding TBE = 0.93 and suggesting excessive geometric strain. These low-TBE complexes likely represent crystallographic artifacts, alternative low-affinity binding modes, or fragment-like molecules lacking elaboration to achieve full binding potential.

**Table A3:** Bottom five ligands ranked by Topological Binding Efficiency.

PDB ID	Target	pK_*d*_	MW (Da)	Sheaf Norm	TBE
4bt5	Unknown	2.76	133	21.2	0.89
3t2q	Kinase	3.66	178	25.6	1.13
4b5w	Enzyme	3.33	89	16.0	1.21
4epy	Protein	3.47	362	41.2	0.93
5j3l	Receptor	4.80	231	29.4	1.41

### Appendix B.3. Full Feature Importance Ranking

Feature importance was assessed via both mean decrease in impurity (MDI) and permutation importance (10 repeats, $R^{2}$-based scoring on the held-out test set). Table A4 reports the complete ranking for all 19 descriptors in the enhanced model. The two methods exhibited strong concordance (Kendall τ=0.798, p<0.001), with both identifying molecular weight and the Sheaf Frobenius norm as the two most influential features. Five of the top ten features by permutation importance were Sheaf-derived descriptors, confirming that topological features contribute substantial predictive information that is not redundant with classical molecular properties. Algebraic connectivity and spectral gap exhibited zero permutation importance, consistent with their near-zero variance among drug-like molecules composed of single connected components.

**Table A4:** Complete feature importance ranking for the 19-descriptor enhanced model, sorted by permutation importance (descending). Permutation importance values represent the mean decrease in test-set $R^{2}$ upon random shuffling of each feature (10 repeats). MDI denotes mean decrease in impurity normalized to sum to unity across all features. Type indicates the descriptor category: Classical (cheminformatics), Sheaf (Sheaf Laplacian spectral), Graph (graph-theoretic), and PH (persistent homology).

Rank	Feature	Type	Perm. Imp.	MDI
1	Molecular weight	Classical	0.155	0.200
2	Sheaf Frobenius norm	Sheaf	0.152	0.190
3	TPSA	Classical	0.119	0.128
4	logP	Classical	0.058	0.102
5	Sheaf trace	Sheaf	0.034	0.048
6	Sheaf eigenvalue std. dev.	Sheaf	0.017	0.059
7	Sheaf max eigenvalue	Sheaf	0.016	0.037
8	Sheaf leading eigenvalue 1	Sheaf	0.015	0.037
9	Mean Betti $\beta_{1}$	PH	0.014	0.052
10	Sheaf leading eigenvalue 2	Sheaf	0.013	0.036
11	Max Betti $\beta_{1}$	PH	0.009	0.048
12	Sheaf mean eigenvalue	Sheaf	0.003	0.016
13	Graph density	Graph	0.002	0.020
14	Average degree	Graph	0.002	0.016
15	Average clustering	Graph	0.001	0.003
16	Sheaf spectral gap	Sheaf	0.000	0.000
17	Sheaf algebraic connectivity	Sheaf	0.000	0.000
18	Mean Betti $\beta_{0}$	PH	<0.001	0.001
19	Number of heavy atoms	Classical	<0.001	0.008

### Appendix B.4. Classical Topological Index Correlation and Benchmark Details

To evaluate the independent information content of Sheaf Laplacian features relative to established graph-theoretic descriptors, Wiener indices and Balaban *J* indices were computed for all 14,041 molecules with valid SMILES representations using RDKit. Table A5 reports the pairwise Pearson correlation coefficients among Sheaf, classical index, and target variables. The Sheaf Frobenius norm exhibited negligible correlation with the Wiener index (r=0.013) but substantial negative correlation with the Balaban *J* index (r=−0.766), the latter reflecting shared but inversely oriented sensitivity to molecular branching.

**Table A5:** Pairwise Pearson correlation coefficients among binding affinity, Sheaf Frobenius norm, molecular weight, Wiener index, and Balaban *J* index (n=14,041). The Sheaf Frobenius norm is essentially orthogonal to the Wiener index (r=0.013) and inversely correlated with the Balaban *J* index (r=−0.766).

	pK_*d*_	Sheaf Frob.	MW	Wiener	Balaban *J*
pK_*d*_	1.000				
Sheaf Frobenius	0.511	1.000			
Molecular weight	0.489	0.953	1.000		
Wiener index	0.006	0.013	0.014	1.000	
Balaban *J*	−0.398	−0.766	−0.756	0.063	1.000

Partial correlation analyses controlling for progressively broader covariate sets are summarized in Table A6. The Sheaf Frobenius norm maintained a substantial partial correlation with binding affinity (r=0.390) even after simultaneously controlling for molecular weight, the Wiener index, and the Balaban *J* index. In contrast, the Wiener index provided no independent predictive signal beyond molecular weight (r=−0.007, p=0.47).

**Table A6:** Partial correlations between molecular descriptors and binding affinity, controlling for progressively broader covariate sets. The Sheaf Frobenius norm retains a substantial partial correlation (r=0.390) with $pK_{d}$ even after controlling for MW, Wiener index, and Balaban *J* simultaneously. The Wiener index carries no independent signal beyond MW.

Partial Correlation	*r*	*p*
*r*(Sheaf, pK_*d*_ | MW)	0.171	<$10^{-70}$
*r*(Sheaf, pK_*d*_ | MW, Wiener)	0.171	<$10^{-70}$
*r*(Sheaf, pK_*d*_ | MW, Wiener, Balaban *J*)	0.390	<$10^{-9}$
*r*(Wiener, pK_*d*_ | MW)	−0.007	0.47
*r*(Balaban *J*, pK_*d*_ | MW)	0.483	<$10^{-9}$

Benchmark Random Forest models were trained under six feature configurations to quantify the incremental predictive contribution of each descriptor group (Table A7). Adding the Wiener index and Balaban *J* to the classical descriptor set yielded a marginal improvement ($\Delta R^{2}=+0.004$), comparable to the gain from adding Sheaf features ($\Delta R^{2}=+0.003$). Combining all descriptor types produced the highest overall performance ($R^{2}=0.451$).

**Table A7:** Benchmark Random Forest performance across feature configurations. All models used identical hyperparameters (200 estimators, max depth 25, $\sqrt{p}$ feature sampling) and identical train-test splits. Adding Wiener/Balaban *J* and Sheaf features to the classical baseline yield comparable gains, and their combination produces the highest overall performance.

Configuration	$n_{\mathrm{feat}}$	$R^{2}$	RMSE	MAE	Spearman ρ
MW only	1	0.282	1.631	1.287	0.524
Classical (no Sheaf)	12	0.425	1.459	1.137	0.634
Sheaf only	9	0.403	1.487	1.162	0.613
Classical + Wiener/Balaban *J*	14	0.450	1.428	1.109	0.652
Classical + Sheaf	19	0.449	1.429	1.113	0.648
Classical + Sheaf + Wiener/Balaban *J*	21	0.451	1.426	1.108	0.653
